# The Arabidopsis COPII components, AtSEC23A and AtSEC23D, are essential for pollen wall development and exine patterning

**DOI:** 10.1093/jxb/ery015

**Published:** 2018-01-30

**Authors:** Mostafa Aboulela, Tsuyoshi Nakagawa, Akinobu Oshima, Kohji Nishimura, Yuji Tanaka

**Affiliations:** 1 Department of Molecular and Functional Genomics, Interdisciplinary Center for Science Research, Shimane University, Matsue, Japan; 2 Bioresources Science, The United Graduate School of Agricultural Sciences, Tottori University, Tottori, Japan; 3 Department of Botany and Microbiology, Faculty of Science, Assiut University, Assiut, Egypt; 4 Department of Biological Science, Faculty of Life and Environmental Science, Shimane University, Matsue, Japan; 5 Department of Applied Bioscience and Biotechnology, Faculty of Life and Environmental Science, Shimane University, Matsue, Japan

**Keywords:** Arabidopsis, thaliana, COPII, elaioplast, ER export, ER–Golgi transport, exine patterning, pollen wall, secretory pathway, tapetal cells, tapetosome

## Abstract

The specialized multilayered pollen wall plays multiple roles to ensure normal microspore development. The major components of the pollen wall (e.g. sporopollenin and lipidic precursors) are provided from the tapetum. Material export from the endoplasmic reticulum (ER) is mediated by coat protein complex II (COPII) vesicles. The *Arabidopsis thaliana* genome encodes seven homologs of SEC23, a COPII component. However, the functional importance of this diversity remains elusive. Here, we analyzed knockout and knockdown lines for *AtSEC23A* and *AtSEC23D*, two of the *A. thaliana* SEC23 homologs, respectively. Single *atsec23a* and *atsec23d* mutant plants, despite normal fertility, showed an impaired exine pattern. Double *atsec23ad* mutant plants were semi-sterile and exhibited developmental defects in pollen and tapetal cells. Pollen grains of *atsec23ad* had defective exine and intine, and showed signs of cell degeneration. Moreover, the development of tapetal cells was altered, with structural abnormalities in organelles. AtSEC23A and AtSEC23D exhibited the characteristic localization pattern of COPII proteins and were highly expressed in the tapetum. Our work suggests that AtSEC23A and AtSEC23D may organize pollen wall development and exine patterning by regulating ER export of lipids and proteins necessary for pollen wall formation. Also, our results shed light on the functional heterogeneity of SEC23 homologs.

## Teaser

Two of the *Arabidopsis thaliana* SEC23 homologs, *AtSEC23A* and *AtSEC23D*, are expressed in the tapetum and play essential roles in pollen wall formation, exine patterning, and tapetum development.

## Introduction

The multilayered pollen wall provides structural and physical support for the microspore cytoplasm, helps the male gametes survive severe environmental conditions, and facilitates pollination and male–female interaction processes (Scott *et al.*, 2004). In *Arabidopsis thaliana*, the pollen wall has an architecturally complex structure comprised of three layers: the inner pectocellulosic-based intine, the outer sporopollenin-based exine, and a lipid-based pollen coat covering the exine. The exine is divided into two layers: nexine and sexine. Further, sexine is subdivided into two structures: pillar-like bacula and roof-like tectum (Ariizumi and Toriyama, 2011). The bacula and the tectum are responsible for the characteristic and taxon-specific architecture of the exine, which is reticulate in *A. thaliana*.

Pollen wall development relies largely on the adjacent sporophytic anther layer, the tapetum, which provides the developing microspores with metabolites, enzymes, nutrients, and structural components necessary to build up the outer exine (Pacini *et al.*, 1985; Blackmore *et al.*, 2007). During the early stages of pollen development, tapetal cells secrete β-1,3-glucanase (callase) to degrade the callose wall around tetrads and release the microspores (Stieglitz, 1977; Verma and Hong, 2001). Then, tapetal cells immediately synthesize lipidic sporopollenin precursors as major components of the exine wall (Quilichini *et al.*, 2014). Finally, during the late stages of pollen development, tapetal cells synthesize and store lipidic materials of the pollen coat in two specialized organelles, the plastid-derived elaioplasts and the endoplasmic reticulum (ER)-derived tapetosomes. Premature or delayed differentiation of tapetal cells, as well as alteration of their internal structure, usually results in defective pollen development and reduced fertility (Kawanabe *et al.*, 2006; Ariizumi and Toriyama, 2011; Liu and Fan, 2013).

The coat protein complex II (COPII)-mediated transport from the ER to the Golgi apparatus represents the first step of the secretory pathway (Schekman and Orci, 1996). Newly synthesized lipids and proteins exit the ER through COPII-coated vesicles heading to the Golgi apparatus for further modification and sorting before reaching their final destinations (Vitale and Denecke, 1999; Foresti and Denecke, 2008). The COPII-coated vesicle formation requires the sequential recruitment of the five cytosolic components, SAR1, SEC23/24, and SEC13/31 (Matsuoka *et al.*, 1998; Lord *et al.*, 2013). In this process, SEC23 interacts with SAR1, SEC24, and SEC31, and functions as a SAR1-specific GTPase-activating protein (GAP) and in cargo recognition with SEC24 (Kuehn *et al.*, 1998; Mancias and Goldberg, 2007; Fromme *et al.*, 2008).

Previous studies have revealed the importance of some conserved amino acids in SEC23. The Arg722 of the yeast SEC23 is necessary for its GAP activity (Bi *et al.*, 2002). In human, a missense mutation at the conserved Phe382 in SEC23A leads to a reduction of its affinity for SEC31 and causes cranio-lenticulo-sutural dysplasia disease (Boyadjiev *et al.*, 2006; Fromme *et al.*, 2007). In *A. thaliana*, an amino acid substitution in AtSEC23A at a conserved aspartic acid has been reported as being essential for the unique interaction with AtSAR1 (Zeng *et al.*, 2015).

Many studies demonstrate the involvement of COPII components in regulating plant growth and development. In *A. thaliana*, we previously reported that both *AtSEC24B* and *AtSEC24C* were redundantly involved in male and female gametophyte development (Tanaka *et al.*, 2013). *AtSEC24A* is essential for male fertility (Conger *et al.*, 2011), ER–Golgi integrity (Faso *et al.*, 2009; Nakano *et al.*, 2009), and maintaining cell size patterning in sepals (Qu *et al.*, 2014). The specific interaction between *AtSAR1* and *AtSEC23A* is required for their function in ER export of proteins (Zeng *et al.*, 2015). More recently, *AtSEC31B* was reported to be required for pollen wall development, probably by regulating the early secretory pathway of tapetal cells (Zhao *et al.*, 2016).

Although SEC23 is an essential component of COPII vesicle formation, its involvement in regulating plant growth and development has yet to be elucidated. Moreover, the functional differences among the SEC23 homologs remain to be determined. In this study, we identified and characterized two *A. thaliana* SEC23 gene homologs, *AtSEC23A* and *AtSEC23D*. Both AtSEC23A and AtSEC23D exhibited the characteristic COPII localization at ER exit sites (ERESs) and are required for proper pollen wall formation, exine patterning, and tapetum development. Our results indicate that ER export of proteins in the early secretory pathway of tapetal cells is a key factor for pollen wall development. Also, this work points to the functional diversity of SEC23 homologs in *A. thaliana*.

## Materials and methods

### Plant materials, growth condition, and transformation

*Arabidopsis thaliana* T-DNA insertion lines (Col-0 background) and the *quartet1* (*qrt1-2*) mutant were obtained from the Arabidopsis Biological Resource Center (ABRC), Ohio State University, USA. *Arabidopsis thaliana* ecotype Col-0 was used as the wild type. The seeds were germinated on a Murashige and Skoog agar medium at 22 °C under continuous light. After 10–14 d, seedlings were transplanted to Jiffy-7 (Jiffy Preforma Production K. K, Yokohama, Japan) and grown at 22 °C under long-day conditions (16 h light/8 h dark) or under continuous light. Transformation of *A. thaliana* was performed by the floral dip method (Clough and Bent, 1998) or the floral inoculating method (Narusaka *et al.*, 2010), and transgenic plants were screened on Murashige and Skoog agar medium containing 100 mg l^−1^ Cefotax (Chugai Pharmaceutical, Tokyo, Japan) and appropriate antibiotics (20 mg l^−1^ hygromycin B or 30 mg l^−1^ kanamycin).

### Genotyping analysis

Genomic DNAs were extracted from leaves following Edwards *et al.* (1991). The T-DNA insertions were analyzed by PCR genotyping with the specific primers GN-*AtSEC23A*-F and GN-*AtSEC23A*-R for *AtSEC23A* or RT-*AtSEC23D*-F and GN-*AtSEC23D*-R for *AtSEC23D*. The T-DNA-LB was used as a common primer for T-DNA detection. The primers are listed in Supplementary Table S1 at *JXB* online.

### Reverse Transcription PCR (RT-PCR) analysis

Total RNAs were extracted from various tissues of wild-type plants using the RNeasy Mini Kit (Qiagen, Tokyo Japan), and used as templates to synthesize cDNAs using ReverTraAce (TOYOBO, Osaka, Japan), according to the manufacturer’s instructions. The *ACTIN2* gene (At3g18780) was used as an internal reference. RT-PCR was performed with 0.2 mg of cDNAs using KOD-Plus-Neo DNA polymerase (TOYOBO) for 26 cycles. The specific primers RT-*AtSEC23A*-F, RT-*AtSEC23A*-R, RT-*AtSEC23D*-F, RT-*AtSEC23D*-R, *ACT2*-F, and *ACT2*-R used for RT-PCR are listed in Supplementary Table S1.

### Preparation of promoter and ORF entry clones

All entry clones shown in Table 1 were prepared with the following method. Promoter and ORF fragments were amplified to add *att*B sequences to their 5' and 3' ends by one or two PCRs with templates and the forward and reverse primers indicated in Table 1. The amplified DNA fragments were introduced into the vectors designated in Table 1 by BP reactions, following the manufacturer’s instructions (Thermo Fisher Scientific, Kanagawa, Japan), to construct the entry clones.

**Table 1.** Materials for construction of entry clones

Entry clone	Cloned DNA fragment	Template	Forward primer^*a*^	Reverse primer^*a*^	Vector
pDONR201-*P*_*AtSEC23A*_ (*att*L1-*P*_*AtSEC23A*_-*att*L2)	*AtSEC23A* promoter (–1996 to –1)^*b*^	*A. thaliana* Col genomic DNA	*P*_*AtSEC23A*_ *-attB*1	*P*_*AtSEC23A*_ *-att*B2	pDONR201
pDONR P4-P1R-*P*_*AtSEC23A*_ (*att*L4-*P*_*AtSEC23A*_-*att*R1)	*AtSEC23A* promoter (–1996 to –1)^*b*^		*P*_*AtSEC23A*_ *-att*B4	*P*_*AtSEC23A*_ *-att*B1r	pDONR P4-P1R
pDONR201-*P*_*AtSEC23D*_ (*att*L1-*P*_*AtSEC23D*_-*att*L2)	*AtSEC23D* promoter (–2126 to –1)^*b*^		*P*_*AtSEC23D*_ *-attB*1	*P*_*AtSEC23D*_ *-att*B2	pDONR201
pDONR P4-P1R-*P*_*AtSEC23D*_ (*att*L4-*P*_*AtSEC23D*_-*att*R1)	*AtSEC23D* promoter (–2126 to –1)^*b*^		*P*_*AtSEC23D*_ *-att*B4	*P*_*AtSEC23D*_ *-att*B1r	pDONR P4-P1R
pDONR P4-P1R-*P*_*nos*_ (*att*L4-*P*_*nos*_-*att*R1)	Nopaline synthase promoter	pGWB 401	*P*_*nos*_ *-att*B4	*P*_*nos*_ *-att*B1r	
pDONR201-*AtSEC23A* (*att*L1-*AtSEC23A*-*att*L2)	*AtSEC23A* ORF^*c*^	pda 01836^*d*^	*AtSEC23A*-*att*B1 (the first PCR) and *att*B1 adaptor (the second PCR)	*AtSEC23A*-*att*B2 (the first PCR) and *att*B2 adaptor (the second PCR)	pDONR201
pDONR201-*AtSEC23D* (*att*L1-*AtSEC23D*-*att*L2)	*AtSEC23D* ORF^c^	pda 04203^*d*^	*AtSEC23D*-*att*B1 (the first PCR) and *att*B1 adaptor (the second PCR)	*AtSEC23D*-*att*B2 (the first PCR) and *att*B2 adaptor (the second PCR)	

^*a*^ Sequences of forward and reverse primers are listed in Supplementary Table S1.

^*b*^ A of the initiation codon is +1.

^*c*^ Nucleotide sequences of ORFs correspond to the region from the translation initiation site to the last amino acid of *AtSEC23A* and *AtSEC23D.*

^*d*^ Arabidopsis full-length cDNA clones, pda01836 and pda04203, were obtained from RIKEN BRC (RIKEN, Tsukuba, Japan).

### Preparation of green fluorescent protein (GFP) fusion constructs for complementation and expression analyses

The pDONRP4-P1R-*P*_*AtSEC23A*_ and pDONR201-*AtSEC23A* entry clones were subjected to LR reaction with R4pGWB550 (Nakagawa *et al.*, 2008) to generate *P*_*AtSEC23A*_*:AtSEC23A-G3GFP*. *P*_*AtSEC23D*_*:AtSEC23D-G3GFP* was prepared by the same procedure using pDONRP4-P1R-*P*_*AtSEC23D*_ and pDONR201-*AtSEC23D* entry clones. In this study, we used G3GFP (Kawakami and Watanabe, 1997), a brighter variant of GFP with S65A/Y145F mutations.

### Staining and semi-thin sectioning

Alexander’s (Alexander, 1969) and DAPI stainings were performed as described in Tanaka *et al.* (2013). For Alexander’s staining, anthers were observed by a BZ-X710 All-in-One fluorescence microscope (KEYENCE, Osaka, Japan). DAPI fluorescence was detected using a BX51 fluorescence microscope (Olympus, Tokyo, Japan) equipped with a UV mirror unit. For the aniline blue staining, buds at the tetrad stage were squeezed into a drop of aniline blue solution (100 mg l^−1^ in 50 mM potassium phosphate buffer, pH 7.5) on a slide and observed using a BX51 fluorescence microscope. For the auramine O staining, pollen grains were immersed in a drop of auramine O solution (0.001% auramine O in 50 mM Tris–HCl, pH 7.5; Dobritsa *et al.*, 2010) on a slide and viewed by confocal microscopy as mentioned below.

Floral buds at different developmental stages were fixed in 4% paraformaldehyde solution, dehydrated in a graded ethanol series, embedded in Technovit 7100 resin (Heraeus Kulzer, Wehrheim, Germany), and sectioned (2–3 µm) by an RV-240 rotary microtome (Yamato Kohki Industrial, Saitama, Japan). Semi-thin sections were stained with a toluidine blue solution (1% toluidine blue, 1% sodium borate) and viewed by the All-in-One fluorescence microscope.

### Promoter:*β-glucuronidase* (*GUS*) assay

For the promoter:*GUS* assays, pDONR201-*P*_*AtSEC23A*_ and pDONR201-*P*_*AtSEC23D*_ entry clones were applied for LR reactions with pGWB233 (Hino *et al.*, 2011) to make *P*_*AtSEC23A*_:*GUS* and *P*_*AtSEC23D*_:*GUS*. These constructs were used for transformation of wild-type *A. thaliana*. T_2_ or T_3_ lines were stained and examined following Nakamura *et al.* (2012).

### *In vitro* pollen germination

Pollen grains of at least six recently opened flowers were tested for germination *in vitro* according to Boavida and McCormick (2007). Images were captured using an SZX16 stereo-microscope (Olympus). Germination rates were calculated by counting at least 500 pollen grains for each sample with ImageJ (http://rsbweb.nih.gov/ij/).

### Transient co-localization analyses in *Nicotiana benthamiana* leaves

The R4 dual-site Gateway cloning system (Aboulela *et al.*, 2017) was used to express two genes in the co-localization analyses. The promoter entry clone pDONRP4-P1R-*P*_*nos*_ and the ORF entry clones pDONR201-*AtSEC23A*, pDONR201-*AtSEC23D*, pDONR201-*AtSEC24A* (Tanaka *et al.*, 2013), and pDONR201-*SYP31* (Tanaka *et al.*, 2013) were used for preparation of the two-gene constructs with R4pDD650-MD8 and R4pGWB6459-MD8 according to Aboulela *et al.* (2017); these were *P*_*nos*_*:AtSEC24A-TagRFP–P*_*nos*_*:AtSEC23A-G3GFP*, *P*_*nos*_*:AtSEC24A-TagRFP–P*_*nos*_*:AtSEC23D-G3GFP*, *P*_*nos*_*: SYP31-TagRFP–P*_*nos*_*:AtSEC23A-G3GFP*, *P*_*nos*_*:SYP31-TagRFP–P*_*nos*_*:AtSEC23D-G3GFP*, and *P*_*nos*_*:AtSEC23A-TagRFP–P*_*nos*_*:AtSEC 23D-G3GFP*.

Transient expression of each two-gene construct in *N. benthamiana* was performed according to the agroinfiltration method described in Shah *et al.* (2013) with some modifications. *Agrobacterium tumefaciens* strain EHA101 was used and the infiltration medium contained 150 mM acetosyringone.

### Confocal microscopy

The fluorescence of G3GFP and TagRFP (red fluorescent protein) was viewed with a TCS SP5 confocal laser scanning microscope (CLSM) (Leica Microsystems, Wetzlar, Germany) using an HCX IRAPO L 25.0 × 0.95 water-immersion objective lens. In observations of anthers, settings described in Huang *et al.* (2013) were applied to distinguish the G3GFP signals from the chlorophyll autofluorescence in anthers. In the co-localization experiments in *N. benthamiana*, G3GFP and TagRFP were excited with the argon (488 nm) or the helium–neon (543 nm) laser lines, and their fluorescence was captured through a band path of 500–530 nm or 555–615 nm, respectively. The fluorescence of auramine O was detected using the fluorescein isothiocyanate settings and an HCX PLAPO 100.0 × 1.40-0.70 oil-immersion objective lens.

### Electron microscopy

SEM observations were performed as described in Tanaka *et al.* (2013) using either an S-4800 field emission scanning electron microscope (Hitachi High-Tech, Tokyo, Japan) or a TM3000 miniscope (Hitachi High-Tech).

Floral buds at appropriate developmental stages were fixed with 2.5% glutaraldehyde in 50 mM sodium phosphate buffer (pH 7.2). The specimens were treated with 1.5% osmium tetroxide in 100 mM cacodylate buffer (pH 7.4) for 90 min and embedded in EPON 812 (TAAB, Berkshire, UK). Ultrathin sections (70 nm) were double-stained with 3% uranyl acetate and lead citrate then observed with a JEM-1400 transmission electron microscope (JEOL, Tokyo, Japan).

## Results

### *AtSEC23A* and *AtSEC23D* are divergent from other Arabidopsis *SEC23* homologs

**Fig. 1.**
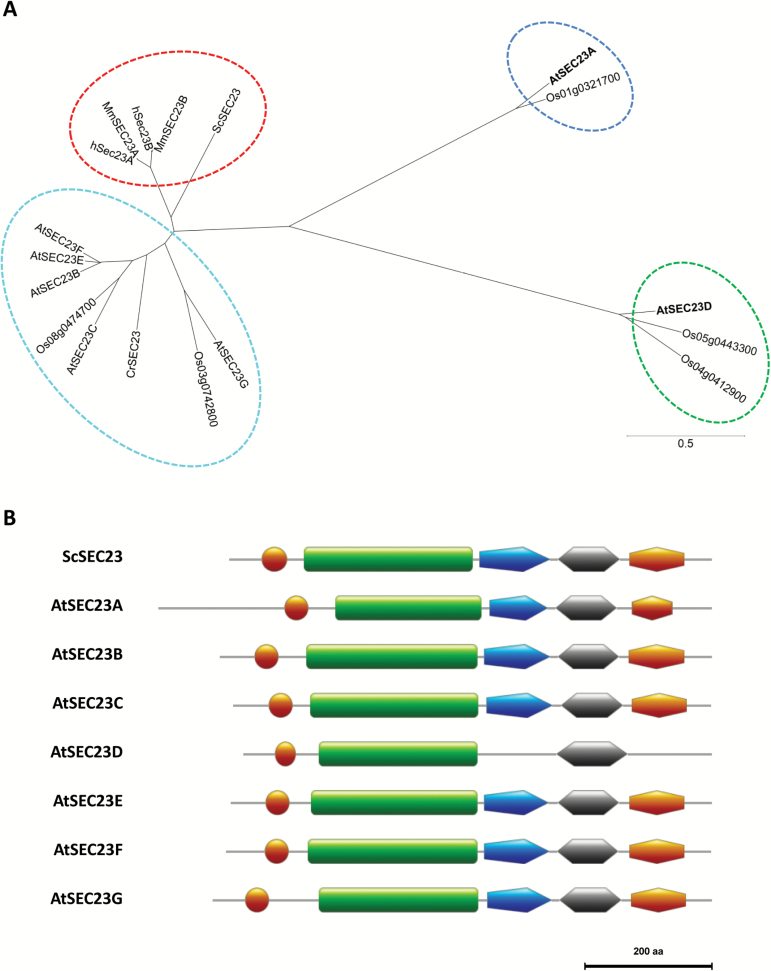
Phylogenetic and domain structure analyses of SEC23 homologs. (A) A phylogenetic tree of SEC23 homologs in plants (*Arabidopsis thaliana* and *Oryza sativa*), green algae (*Chlamydomonas reinhardtii*), mammals (*Homo sapiens* and *Mus musculus*), and yeast (*Saccharomyces cerevisiae*). Amino acid sequences were aligned using the Neighbor–Joining method and bootstrap values of 1000 replications by the ClustalW ver. 1.83 program (http://clustalw.ddbj.nig.ac.jp/). The tree was drawn by GENETYX-Tree software (Genetyx, Tokyo, Japan) based on the alignment. The scale bar demonstrates the evolutionary distance. Accession nos: AtSEC23A (*A. thaliana*, NP_567217), AtSEC23B (NP_563741), AtSEC23C (NP_179757), AtSEC23D (NP_565651), AtSEC23E (NP_189008), AtSEC23F (NP_193152), AtSEC23G (NP_568626), Os01g0321700 (*O. sativa*, XP_015621728), Os03g0742800 (AAR87299), Os04g0412900 (XP_015636882), Os05g0443300 (BAF17587), Os08g0474700 (XP_015650237), CrSEC23 (*C. reinhardtii*, XP_001702936), hSec23A (*H. sapiens*, CAA65774), hSec23B (CAA65775), MmSEC23A (*M. musculus*, NP_033173), MmSEC23B (NP_062761), and ScSEC23 (*S. cerevisiae*, NP_015507). (B) Structure of SEC23 domains in yeast and *A. thaliana*. The domains were predicted with the motif database Pfam (http://pfam.xfam.org/) and drawn using the image creator MyDomains (http://prosite.expasy.org/mydomains/). The five predicted domains are indicated as follows: circle, zinc finger; rectangle, trunk; pentagon, β-barrel; first hexagon, all-helical; and second hexagon, gelsolin-like.

Within the *A. thaliana* genome, there are five homologs for SAR1, seven for SEC23, three for SEC24, and two for each of SEC12, SEC13, SEC16, and SEC31 (Robinson *et al.*, 2007; Chung *et al.*, 2016). The seven SEC23 homologs, At4g01810, At1g05520, At2g21630, At2g27460 At3g23660, At4g14160, and At5g43670, were named AtSEC23A, AtSEC23B, AtSEC23C, AtSEC23D, AtSEC23E, AtSEC23F, and AtSEC23G, respectively, following Chung *et al.* (2016). Phylogenetic analysis of SEC23 homologs in yeast, green algae, plants, and mammals showed that five of the *A. thaliana* SEC23 homologs were clustered in a single clade with rice and green algae homologs (Fig. 1A). While both AtSEC23A and AtSEC23D were divergent from other AtSEC23 proteins, each was separated in a distinct clade with only homologs of rice. AtSEC23A and AtSEC23D showed no significant identity with other AtSEC23s (Supplementary Fig. S1).

In *Saccharomyces cerevisiae*, ScSEC23 includes five domains, zinc finger, trunk, β-barrel, all-helical, and gelsolin-like domains (Bi *et al.*, 2002). These five domains are well conserved within AtSEC23s, except for AtSEC23D which is missing the β-barrel and gelsolin-like domains (Fig. 1B). Sequence alignment of AtSEC23s and ScSEC23 revealed that only AtSEC23A and AtSEC23D have an amino acid substitution at the conserved aspartate residue in the trunk domain [Asp351 of ScSEC23 to Cys484 of AtSEC23A (Zeng *et al.*, 2015) or Glu337 of AtSEC23D] (Supplementary Fig. S1). Interestingly, AtSEC23D is also missing Phe366 [equivalent to Phe382 of human SEC23A (Boyadjiev *et al.*, 2006; Fromme *et al.*, 2007)] and the catalytic Arg706 [Arg722 of ScSEC23 (Bi *et al.*, 2002)], which are conserved in ScSEC23 and other AtSEC23s (Supplementary Fig. S1). These results indicated that both AtSEC23A and AtSEC23D are distinct from other AtSEC23s.

### *atsec23a* and *atsec23d* single mutants show defects in exine patterning

**Fig. 2.**
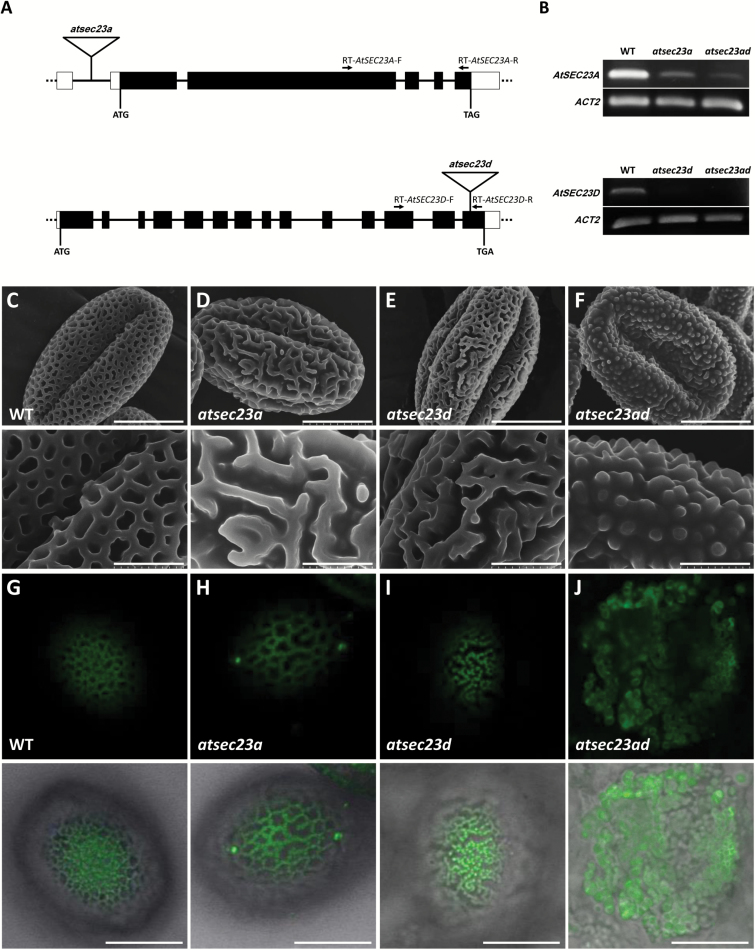
SEM analysis of pollen surface structures in the wild type (WT), *atsec23a*, *atsec23d*, and *atsec23ad*. (A) Schematic diagrams of *AtSEC23A* (upper) and *AtSEC23D* (lower) genes and T-DNA insertion sites. Black boxes, white boxes, and black solid lines represent the exons, untranslated regions, and introns, respectively. Triangles and arrows indicate the T-DNA insertions and positions of primers for RT-PCR analyses, respectively. (B) Expression analysis of *AtSEC23A* and *AtSEC23D* by RT-PCR in WT, *atsec23a*, *atsec23d*, and *atsec23ad*. *Actin2* was used as an internal reference. (C–F) SEM images showing pollen surface structures of the WT (C), *atsec23a* (D), *atsec23d* (E), and *atsec23ad* (F). Lower panels indicate magnified images of the pollen surface structures in the upper panels. (G–J) Auramine O staining of the WT (G), *atsec23a* (H), *atsec23d* (I), and *atsec23ad* (J). Upper panels and lower panels show the fluorescence images of auramine O and merged images of the auramine O fluorescence and the bright field, respectively. Scale bars=10 µm (C–F; upper panels and G–J) and 3 µm (C–F; lower panels).

To understand the functions of AtSEC23s during plant development, we prepared homozygous T-DNA insertion lines of the seven *AtSEC23* genes and screened them for developmental abnormalities. Among them all, we found abnormalities in the pollen wall structure of insertion lines of *AtSEC23A* (At4g01810) and *AtSEC23D* (At2g27460) (Fig. 2; Supplementary Fig. S2). These T-DNA insertion lines, SALK_021996 and SALK_012411 (Alonso *et al.*, 2003), were named *atsec23a* and *atsec23d*, respectively, and used for further experiments. In *atsec23a* and *atsec23d*, T-DNAs were inserted in the first intron located at the 5'-untranslated region and in the last (14th) exon of *AtSEC23A* and *AtSEC23D*, respectively (Fig. 2A). RT-PCR analysis revealed that *atsec23a* and *atsec23d* were knockdown and knockout mutants, respectively (Fig. 2B). In contrast to wild-type pollen grains which had an exine with the characteristic net-like structure (Fig. 2C), the pollen grains of *atsec23a* and *atsec23d* showed an abnormal exine phenotype represented by a partial loss of the net-like structure and incomplete tectum formation in some areas (Fig. 2D, E), which was also shown by auramine O (exine-specific fluorescent dye) stain (Fig. 2G–I). A complementation test was conducted by introducing *P*_*AtSEC23A*_*:AtSEC23A-G3GFP* and *P*_*AtSEC23D*_*:AtSEC23D-G3GFP* into *atsec23a* and *atsec23d*, respectively. Several complemented lines showing normal exine patterning were obtained (Supplementary Fig. S2F, G). These results indicated that the depletion of AtSEC23A or AtSEC23D leads to defects in the exine patterning.

**Fig. 3.**
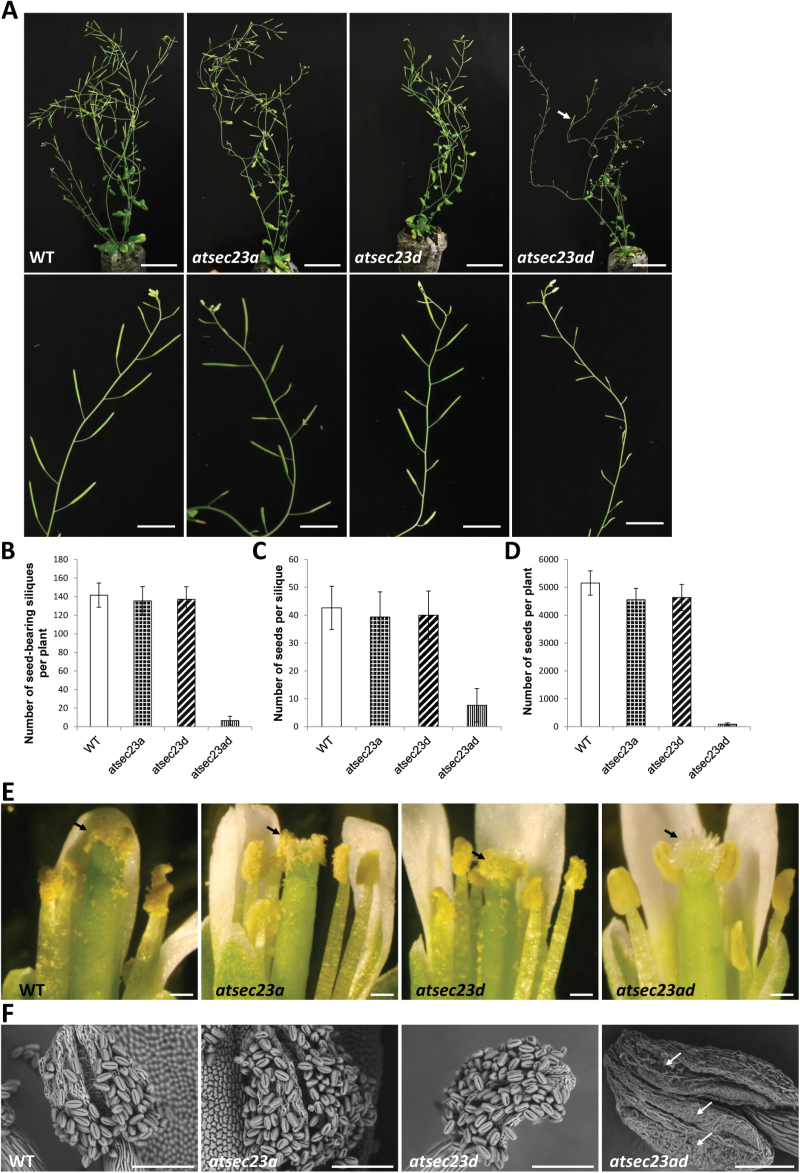
Fertility analysis of *atsec23a*, *atsec23d*, and *atsec23ad*. (A) Two-month-old WT, *atsec23a*, *atsec23d*, and *atsec23ad*. The arrow indicates one of the few seed-bearing siliques. The lower panels show a branch of each of the WT, *atsec23a*, *atsec23d*, and *atsec23ad*. (B–D) Seed and silique set analyses in *atsec23a*, *atsec23d*, and *atsec23ad*. The analyses include the number of seed-bearing siliques per plant (B; *n*=13), the number of seeds per silique (C; *n*=40), and the number of seeds per plant (D; *n*=10). Error bars indicate the SD. (E) Dissected flowers of *atsec23a*, *atsec23d*, and *atsec23ad*. Arrows show stigmas with or without pollen grains. (F) SEM micrographs of dehiscent anthers in the WT, *atsec23a*, *atsec23d*, and *atsec23ad*. Arrows indicate aggregates of collapsed pollen grains adhering to the anther wall. Scale bars=5 cm (A; upper panels), 2 cm (A; lower panels), 200 µm (E), and 100 µm (F).

To analyze functional redundancy of *AtSEC23A* and *AtSEC23D*, we generated the double mutant *atsec23ad* by cross-fertilization. Most pollen grains in the *atsec23ad* became collapsed and flattened (Fig. 2F, J), and tended to aggregate as a bulk of remnant materials strongly adhesive to anther walls failing to be released (Fig. 3F). Many granules of different shape and size were irregularly scattered on the pollen surface (Fig. 2F). These granules showed auramine O fluorescence, indicating that they are composed of a material similar to sporopollenin (Fig. 2J). Therefore, these results suggested that *AtSEC23A* and *AtSEC23D* contribute to exine formation and sporopollenin deposition.

### The *atsec23ad* double mutant shows a semi-sterile phenotype with impaired function of male gametophytes

Because *atsec23ad* showed severe pollen defects, we analyzed the seed production after self-pollination. The wild type, *atsec23a*, and *atsec23d* contained elongated siliques with an almost full set of seeds (Fig. 3A–C), and the total seed yield was ~5000 seeds per plant (Fig. 3D). In contrast, the *atsec23ad* mutant showed a semi-sterile phenotype with <100 seeds per plant (Fig. 3D). Most siliques were short and devoid of any seeds, and only a few siliques contained a reduced set of developing seeds (Fig. 3A, C).

Next, we observed open flowers. Stamens of *atsec23a*, *atsec23d*, and *atsec23ad* were comparable in height with those of the wild type (Fig. 3E), and their anthers showed normal dehiscence (Fig. 3F). In the wild type, *atsec23a*, and *atsec23d*, an abundant amount of pollen grains was visible on anthers and adhered to stigmas, whereas no pollen grains appeared on *atsec23ad* anthers or stigmas (Fig. 3E). To determine whether *atsec23ad* also has defects in the female gametophyte, we cross-pollinated stigmas of *atsec23ad* with wild-type pollen grains. Normal elongated siliques with an almost full set of seeds indistinguishable from that of the wild type were produced (Supplementary Fig. S3), indicating that female gametophytes function normally in *atsec23ad*. The insufficiency of both AtSEC23A and AtSEC23D is linked to defects in male gametophytes and results in the semi-sterile phenotype.

### The *atsec23a* single mutant exhibits an impaired pollen germination

**Fig. 4.**
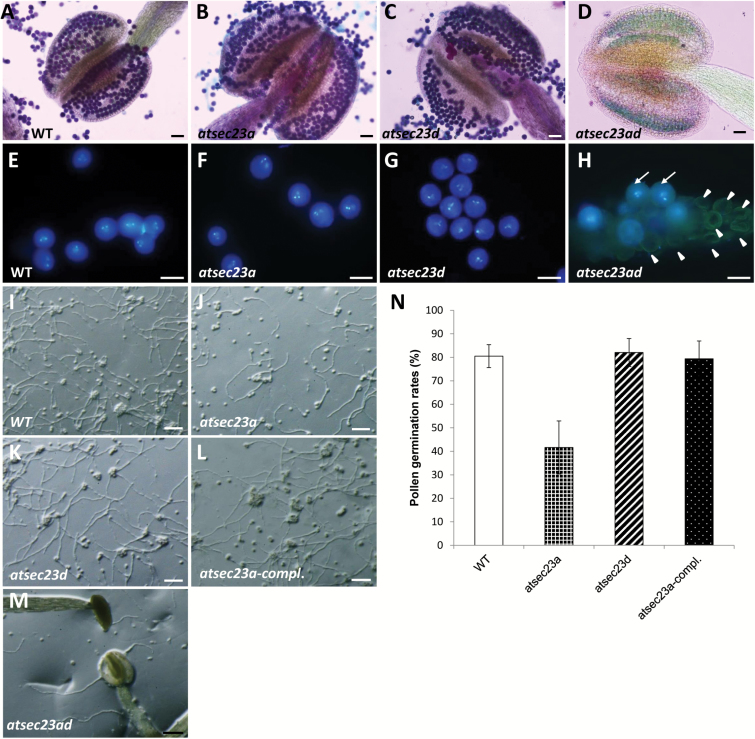
Phenotypic analyses of WT, *atsec23a*, *atsec23d*, and *atsec23ad* pollen grains. (A–D) Alexander’s staining of WT (A), *atsec23a* (B), *atsec23d* (C), and *atsec23ad* pollen grains (D). (E–H) DAPI staining of mature pollen grains in the WT (E), *atsec23a* (F), *atsec23d* (G), and *atsec23ad* (H). Arrows and arrowheads indicate the few normally developed pollen grains with three nuclei and non-stained pollen grains, respectively. (I–M) Light microscopy images of pollen germination in the WT (I), *atsec23a* (J), *atsec23d* (K), *atsec23a-compl.* (L), and *atsec23ad* (M). (N) Pollen germination rate in the WT, *atsec23a*, *atsec23d*, and *atsec23a-compl*. Error bars indicate the SD. Scale bars=50 µm (A–D), 20 µm (E–H), and 200 µm (I–M).

To confirm whether the semi-sterility in *atsec23ad* was caused by an abnormal pollen production, we examined the viability of pollen grains by Alexander’s and DAPI stainings. The wild-type, *atsec23a*, and *atsec23d* anthers contained purple-stained (viable) pollen grains (Fig. 4A–C). In contrast, most anthers of the *atsec23ad* mutant contained green-stained (dead) pollen grains (Fig. 4D), and only a few purple-stained pollen grains were observed in some anthers (Supplementary Fig. S4A), consistent with the production of few seeds in *atsec23ad*. DAPI staining showed that the wild type, *atsec23a*, and *atsec23d* generated pollen grains containing two sperm nuclei and one vegetative nucleus, indicating normal development and male mitosis (Fig. 4E–G). In contrast, the majority of pollen grains generated in *atsec23ad* were smaller and collapsed with no DAPI signals; however, only a few pollen grains possessed the three nuclei (Fig. 4H), consistent with Alexander’s staining results.

To examine whether the phenotype of *atsec23a*, *atsec23d*, and *atsec23ad* is attributed to gametophytic or sporophytic defects, we performed genetic analyses using heterozygous lines. Almost all pollen grains generated from heterozygous lines of both +/*atsec23a* and +/*atsec23d* were indistinguishable from the wild-type pollen grains (Supplementary Fig. S5A–F), indicating that the incomplete exine pattern was caused by defects in sporophytic tissues. Furthermore, we observed pollen grains produced by a heterozygous line of *atsec23a* in the homozygous *atsec23d* and *qrt1-2* background (+/*atsec23a*, *atsec23d*/*atsec23d*, *qrt1-2*/*qrt1-2*). *qrt1-2* causes incomplete separation of microspores in tetrads, enabling precise segregation analysis of pollen grains derived from a single microsporocyte (Preuss *et al.*, 1994). As revealed by SEM and Alexander’s staining, although the exine pattern showed the phenotype of *atsec23d*/*atsec23d*, most pollen tetrads had four uncollapsed pollen grains similar to those from *qrt1-2* (Supplementary Fig. S5G–O), indicating that the failure of pollen development in *atsec23ad* was due to a sporophytic aberration. These results suggested that both *AtSEC23A* and *AtSEC23D* function sporophytically.

Next, we performed an *in vitro* pollen germination assay. Pollen grains of *atsec23d* showed a germination rate of 82% comparable with the wild-type pollen grains (80.5%), while only 41.6% of *atsec23a* pollen grains could germinate (Fig. 4I–K, N). The quantitative analysis of pollen germination for *atsec23ad* was difficult because most pollen grains were collapsed and adhesive to anther walls. However, after forcing pollen grains to separate from anther walls using forceps, very few germinated pollen grains were seen on the germination medium or within the anther (Fig. 4M). Complemented *atsec23a* lines carrying *P*_*AtSEC23A*_*:AtSEC23A-G3GFP* showed similar pollen germination rates to that of the wild type (Fig. 4L, N). These results indicated that pollen germination was normal in *atsec23d* but impaired in *atsec23a*, and also showed the presence of a few functional pollen grains in *atsec23ad*, which agreed with the few seeds obtained from *atsec23ad*. Therefore, these findings indicated that *atsec23a* produces viable pollen grains that normally undergo pollen mitosis but with a reduced germination.

### *AtSEC23A* and *AtSEC23D* are highly expressed in the tapetum

**Fig. 5.**
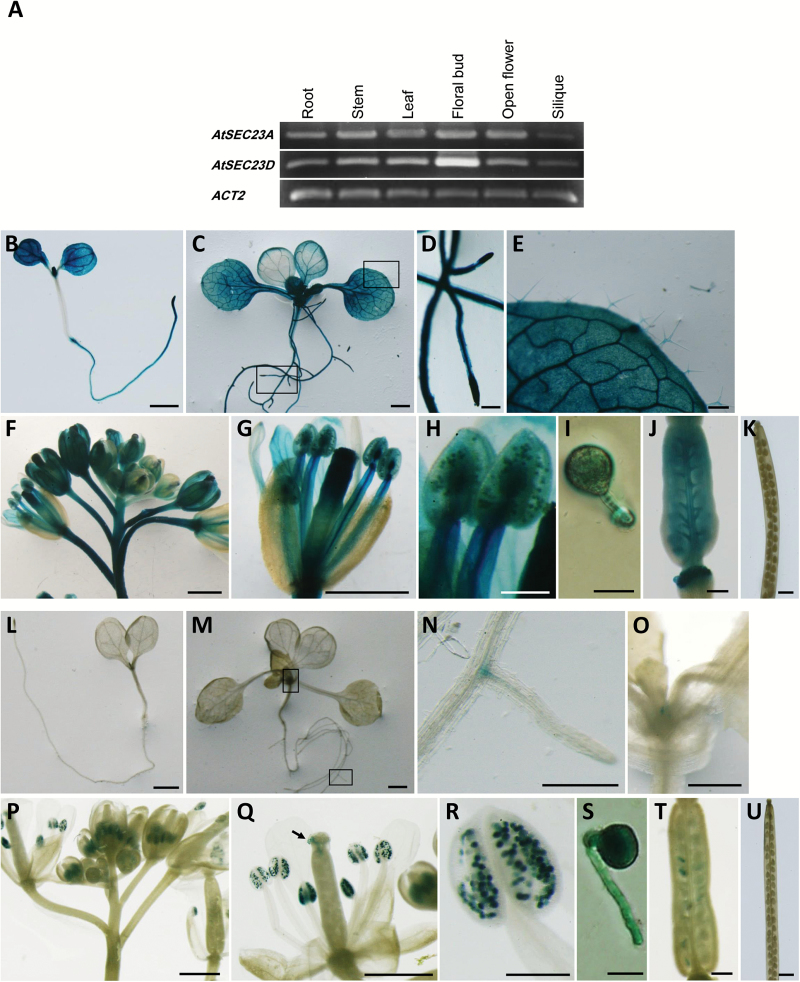
Expression patterns of *AtSEC23A* and *AtSEC23D* in *A. thaliana.* (A) RT-PCR analysis of *AtSEC23A* and *AtSEC23D* in root, stem, leaf, floral bud, open flower, and silique of the WT. *Actin2* was monitored as an internal reference. (B–U) GUS staining of transgenic *A. thaliana* carrying *P*_*AtSEC23A*_*:GUS* (B–K) or *P*_*AtSEC23D*_*:GUS* (L–U). Five-day-old seedlings (B, L), 14-day-old seedlings (C, M), inflorescences (F, P), flowers (G, Q), anthers (H, R), germinated pollen grains (I, S), fertilized ovaries (J, T), and mature siliques (K, U). (D, E) and (N, O) are magnified images of the boxed areas in (C) and (M), respectively. The arrow in (Q) indicates germinated pollen grains on stigmatic papillae. Scale bars=1 mm (B, C, F, G, K, L, M, P, Q, and U), 200 µm (D, E, H, J, N, O, R, and T), and 20 µm (I and S).

Expression of *AtSEC23s* was anticipated to be universally distributed throughout the whole plant by GENEVESTIGATOR (Robinson *et al.*, 2007). In this study, we analyzed the expression pattern of *AtSEC23A* and *AtSEC23D* in detail. By RT-PCR, *AtSEC23A* was expressed almost equally in all investigated organs except siliques, which showed a weak expression (Fig. 5A). *AtSEC23D* was expressed in all investigated organs but was most abundant in closed floral buds, and weak in siliques (Fig. 5A). In the promoter:*GUS* assay, GUS activity in transgenic plants carrying *P*_*AtSEC23A*_*:GUS* was widely detected in flower parts including sepals, petals, filaments, anther walls, mature pollen grains, pollen tubes, and young siliques (Fig. 5F–J), but not in mature siliques (Fig. 5K). In 5-day-old seedlings, GUS activity was detected in the whole seedling except the hypocotyl (Fig. 5B). In 14-day-old seedlings, GUS activity was observed throughout the whole plant including roots, cotyledons, true leaves, trichomes, and leaf primordia (Fig. 5C–E). In transgenic plants carrying *P*_*AtSEC23D*_*:GUS*, the GUS activity was detected mainly in floral buds and open flowers (Fig. 5P, Q), mature pollen grains, pollen tubes (Fig. 5Q–S), and fertilized ovules (Fig. 5T), but was not observed in other floral parts including sepal, petal, filaments, anther walls (Fig. 5P–R), and mature siliques (Fig. 5U). In 5-day-old seedlings, no expression was detected (Fig. 5L). In 14-day-old seedlings, only a negligible GUS staining was observed in roots and leaf primordia (Fig. 5M–O).

**Fig. 6.**
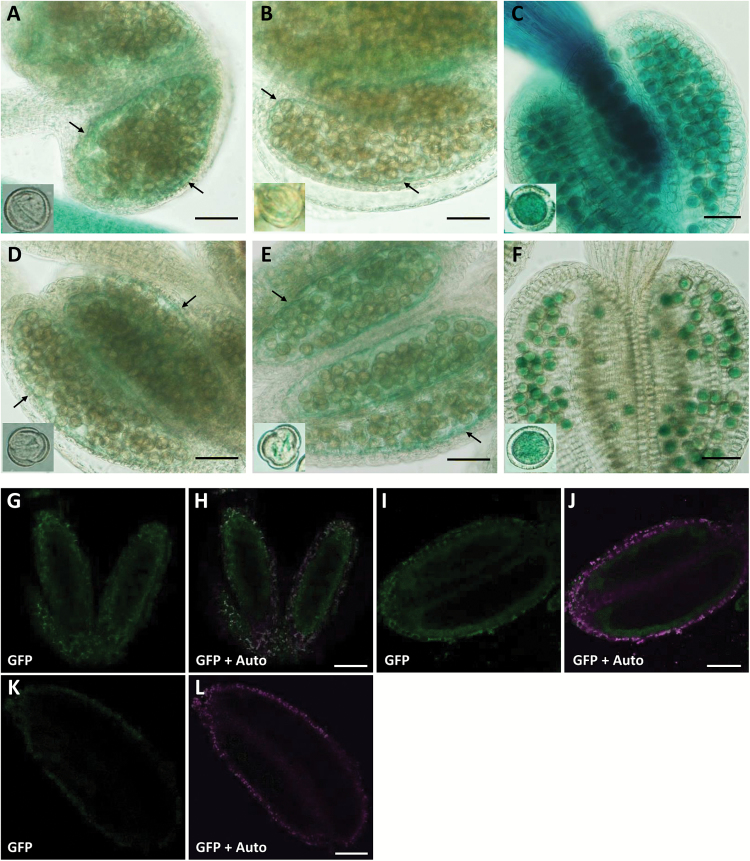
Detailed expression patterns of *AtSEC23A* and *AtSEC23D* in anthers. (A–F) GUS activity in the tapetum and pollen grains of transgenic *A. thaliana* carrying *P*_*AtSEC23A*_*:GUS* (A–C) or *P*_*AtSEC23D*_*:GUS* (D–F). GUS staining of anthers at uninucleate (A, D), bicellular (B, E), and tricellular stages (C, F). Insets are magnified images of pollen grains. Arrows indicate the tapetum. (G–J) GFP fluorescence in the tapetum at the uninucleate stage of transgenic *A. thaliana* carrying *P*_*AtSEC23A*_*:AtSEC23A-G3GFP* (G, H) or *P*_*AtSEC23D*_*:AtSEC23D-G3GFP* (I, J). (G–L) GFP fluorescence in the tapetum at the uninucleate stage of transgenic *A. thaliana* carrying *P*_*AtSEC23A*_*:AtSEC23A-G3GFP* (G, H) or *P*_*AtSEC23D*_*:AtSEC23D-G3GFP* (I, J), and the WT as a reference (K, L). GFP, signal of G3GFP; Auto, autofluorescence of chlorophyll. Scale bars=40 µm.

Next, we followed the expression closely in anthers. GUS signals were observed predominantly in tapetal cells at uninucleate and bicellular stages in *P*_*AtSEC23A*_*:GUS* and *P*_*AtSEC23D*_*:GUS* transgenic plants; meanwhile, no or few signals were detected in developing microspores (Fig. 6A, B, D, E). At the tricellular stage, when tapetum degeneration has completed, strong GUS signals were observed in mature pollen grains (Fig. 6C, F). We further traced the expression of both genes in tapetal cells of complemented lines, *atsec23a* carrying *P*_*AtSEC23A*_*:AtSEC23A-G3GFP* and *atsec23d* carrying *P*_*AtSEC23D*_*:AtSEC23D-G3GFP*. In both cases, GFP fluorescence was detected mainly in the tapetum at the uninucleate stage (Fig. 6G–J). Taken together, although *AtSEC23A* and *AtSEC23D* show different expression patterns in the whole plant, both are highly expressed in the tapetum.

### AtSEC23A and AtSEC23D are localized to the cytoplasm and ERESs

**Fig. 7.**
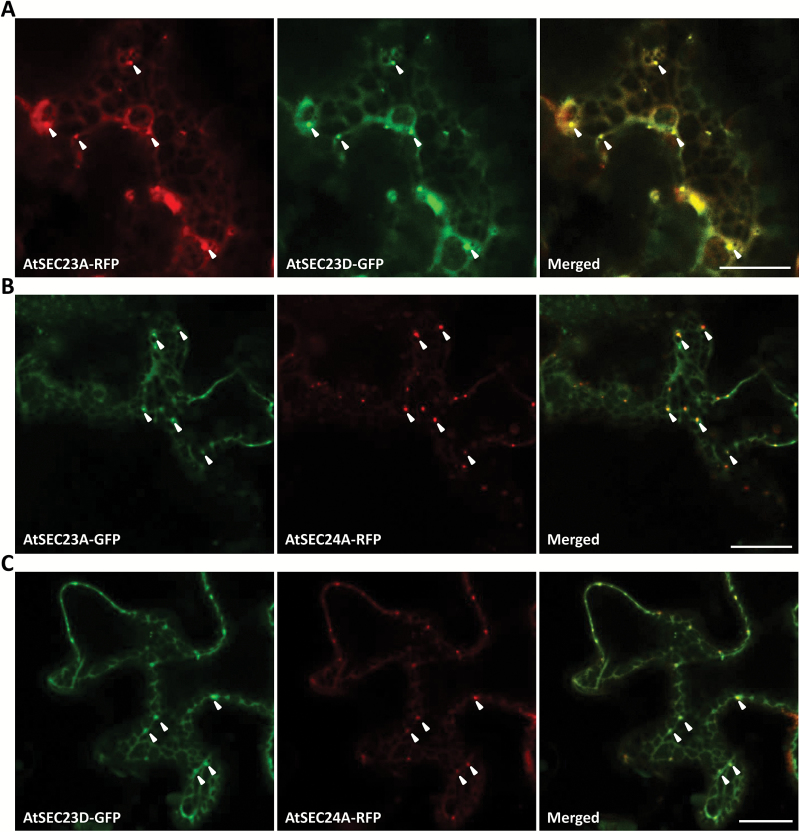
Intracellular localization of AtSEC23A and AtSEC23D in *N. benthamiana* leaf epidermal cells. (A) Fluorescent images of an epidermal cell co-expressing AtSEC23D–G3GFP and AtSEC23A–TagRFP. (B, C) Fluorescent images of epidermal cells co-expressing AtSEC23A–G3GFP (B) or AtSEC23D–G3GFP (C) with the ERES marker AtSEC24A–TagRFP. Arrowheads indicate ERESs. Scale bars=20 µm.

AtSEC23A has shown ERES localization when co-expressed with SAR1A in *A. thaliana* protoplast (Zeng *et al.*, 2015). To examine the subcellular localization of AtSEC23D, we transiently co-expressed AtSEC23D–G3GFP with AtSEC23A–TagRFP in *N. benthamiana* leaves. AtSEC23D–G3GFP exhibited a similar localization pattern to AtSEC23A–TagRFP; both were localized to the cytoplasm, with the existence of many dot-like structures (Fig. 7A; Supplementary Fig. S6A). To confirm that these dot-like structures were ERESs, we used the ERES marker AtSEC24A (Hanton *et al.*, 2007; Wei and Wang, 2008) and the *cis*-Golgi marker SYP31, which is transported as a cargo to the Golgi apparatus via the secretory pathways (Bubeck *et al.*, 2008). AtSEC24A–TagRFP was co-localized with AtSEC23A–G3GFP and AtSEC23D–G3GFP, and the dot-like structures overlapped with the ERESs labeled by AtSEC24A–TagRFP (Fig. 7B, C; Supplementary Fig. S6B, C). Furthermore, a time-lapse analysis showed that these GFP and RFP signals moved together as a single unit in the cytoplasm (Supplementary Movies S1, S2), confirming that these dots were ERESs. In addition, RFP signal of SYP31–TagRFP was detected in close proximity to the ERESs to which AtSEC23A–G3GFP and AtSEC23D–G3GFP were localized (Supplementary Fig. S7A, B). These results indicated that both AtSEC23A and AtSEC23D exhibit the characteristic localization of COPII, suggesting that they contribute to COPII formation and ER–Golgi vesicle transport.

### The significant defects in microspore development of the *atsec23ad* double mutant appear at the late uninucleate stage

**Fig. 8.**
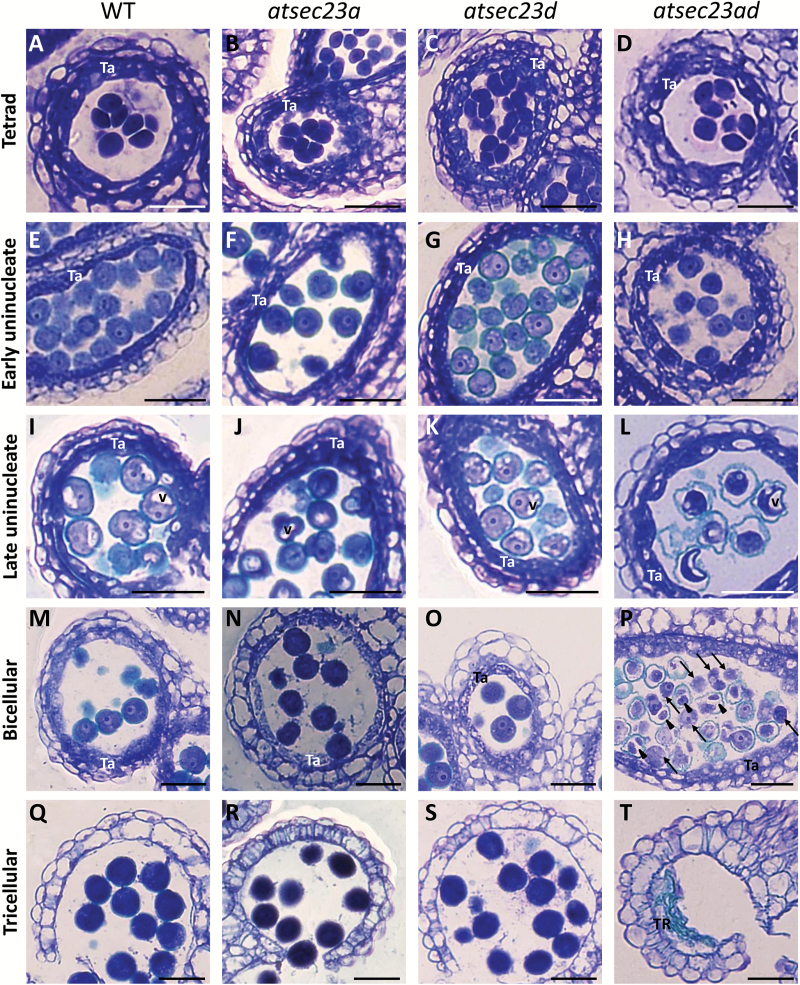
Semi-thin sections of anthers at different developmental stages. Sections of anthers at tetrad (A–D), early uninucleate (E–H), late uninucleate (I–L), bicellular (M–P), and tricellular stages (Q–T). Arrowheads and arrows indicate microspores with detached pollen walls and without pollen walls, respectively. Ta, tapetum; TR, tapetum residue; V, vacuole. Scale bars=30 µm.

To determine the precise timing of pollen defects, we examined callose wall deposition on tetrads by aniline blue staining. Tetrads of *atsec23a*, *atsec23d*, and *atsec23ad* showed levels of fluorescence comparable with that of the wild type (Supplementary Fig. S8), suggesting that the defects were later than this stage. Next, we analyzed the microspore development from the tetrad to the tricellular stage by semi-thin sectioning. The wild type, *atsec23a*, *atsec23d*, and *atsec23ad* showed no obvious differences in microspore development at the tetrad stage (Fig. 8A–D); however, tapetum was more vacuolated in *atsec23ad* than in the wild type, *atsec23a*, and *atsec23d* (Fig. 8D). At the early uninucleate stage, microspores were normally released from the tetrads in wild-type, *atsec23a*, *atsec23d*, and *atsec23ad* anthers (Fig. 8E–H). At the late uninucleate stage, microspores and tapetal cells of *atsec23a* and *atsec23d* showed comparable development with that of the wild type (Fig. 8I–K). In contrast, most microspores in *atsec23ad* had irregular shapes with abnormal exine walls that mostly detached from the microspore cells (Fig. 8L). At the bicellular stage, microspore development proceeded in wild-type, *atsec23a*, and *atsec23d* anthers with no detectable differences (Fig. 8M–O). In contrast, the defects of *atsec23ad* became more obvious, with a majority of pollen grains misshapen and shrunken, and displaying signs of degeneration (Fig. 8P). Many pollen grains have shown a clear separation of the vegetative and the generative cells with detached exine walls (Fig. 8P; arrowheads), while others had no visible exine walls (Fig. 8P; arrows). The tapetum degeneration was initiated normally in the *atsec23a* and *atsec23d* as in wild-type anthers (Fig. 8M–O), while metabolically active swollen tapetal cells with many vacuoles were evident in the *atsec23ad* anthers, indicating a delayed programmed cell death (Fig. 8P). At the tricellular stage, the pollen mitosis II was completed, and numerous tricellular pollen grains were found in wild-type, *atsec23a*, and *atsec23d* anther locules (Fig. 8Q–S). In contrast, only some remnants of tapetal cells, pollen materials, and empty pollen walls were adherent to locule walls in most of the *atsec23ad* anthers (Fig. 8T), and only a few intact pollen grains were occasionally seen in some anther locules (Supplementary Fig. S4B). These observations indicated that *atsec23a* and *atsec23d* showed no detectable defects in this analysis, whereas *atsec23ad* mutants were defective in exine wall formation, which directly affected the whole process of pollen development and subsequently the seed yield.

### Exine and intine formation is impaired in the *atsec23ad* double mutant

**Fig. 9.**
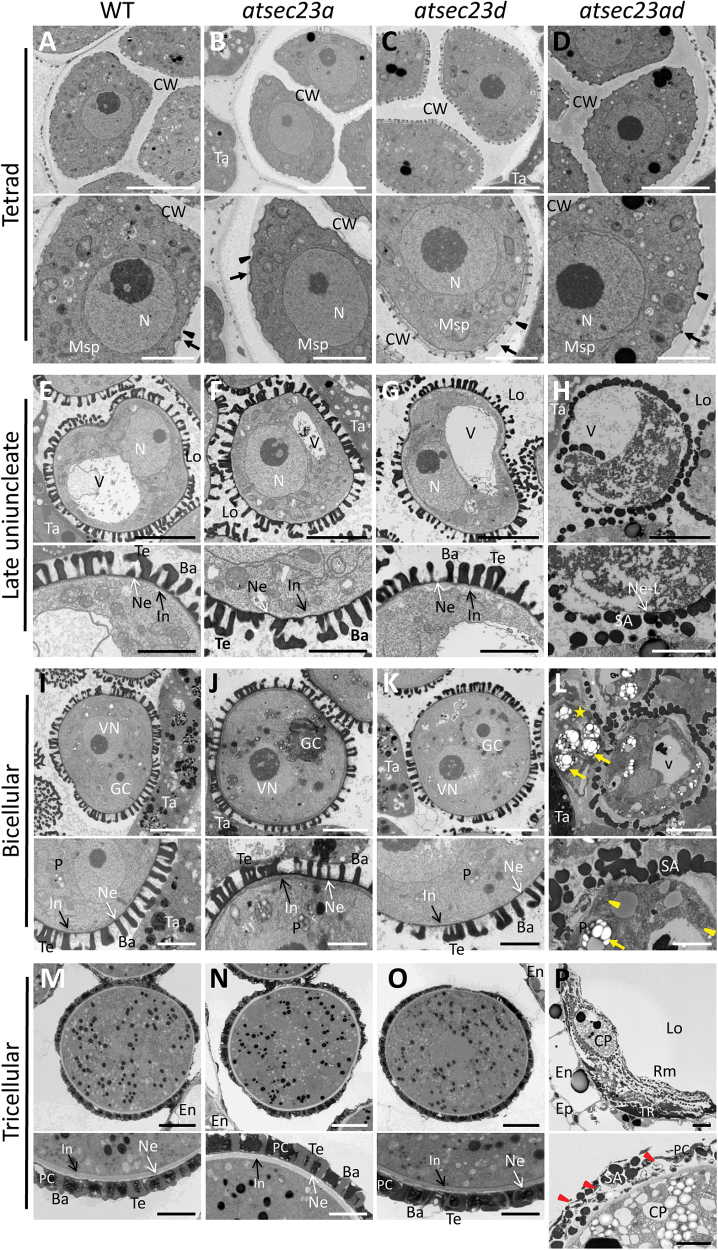
TEM micrographs of microspores at different developmental stages. (A–D) Ultrastructure of microspores at the tetrad stage. Arrowheads and arrows label the primexine and protecta, respectively. (E–H) Ultrastructure of microspores at the late uninucleate stage. (I–L) Ultrastructure of pollen grains at the bicellular stage. A star, arrows, and arrowheads indicate a naked microspore with no walls, plastids with abnormal morphology, and mitochondria, respectively. (M–P) Ultrastructure of pollen grains at the tricellular stage. Arrowheads show the irregular electron-lucent structures in the pollen coat of *atsec23ad*. Lower panels are magnifications of pollen surface structures in the upper panels. Ba, baculum; CP, collapsed pollen grains; CW, callose wall; En, endodermis; Ep, epidermis; GC, generative cell; In, intine; Lo, locule; Msp, microspore; N, nucleus; Ne, nexine; Ne-L, nexine-like structure; P, plastid; PC, pollen coat; Rm, remnants of pollen materials; SA, sporopollenin-like aggregations; Ta, tapetum; Te, tectum; TR, tapetum residue; V, vacuole; VN, vegetative nucleus. Scale bars=5 µm in upper panels, and 2 µm in lower panels.

We further analyzed the ultrastructure of microspores and tapetal cells by TEM. In the wild type at the tetrad stage, microspores enveloped in callose wall showed successful plasma membrane undulation and primexine synthesis, followed by probacula deposition (Fig. 9A). Similar processes were observed in *atsec23a*, *atsec23d*, and *atsec23ad* (Fig. 9B–D). At the late uninucleate stage, the wild type, *atsec23a*, and *atsec23d* developed a well-defined bacula and tectum, and a thin layer of nexine (Fig. 9E–G). In addition, a primary intine was seen at this stage (Fig. 9E–G; black arrows). In *atsec23ad* microspores, such typical exine structures were completely missing. Instead, the microspores were surrounded by a fragmented nexine-like layer and many electron-dense semi-spherical sporopollenin-like aggregations (Fig. 9H). The cytoplasm of most microspores showed signs of degeneration with detached pollen walls. At the bicellular stage, wild-type, *atsec23a*, and *atsec23d* microspores continued their development with an expanded bacula and thicker tectum (Fig. 9I–K). In addition, the intine became more obvious at this stage (Fig. 9I–K; arrows). In contrast, the *atsec23ad* microspores showed more damaged pollen walls in which the sporopollenin-like aggregations increased in size and fused together with clear separation from the microspore plasma membrane (Fig. 9L). Some pollen grains were seen as naked cells with no walls (Fig. 9L;Supplementary Fig. S4C; yellow stars), while others had only empty walls without microspore cells (Supplementary Fig. S4C; yellow asterisks), consistent with semi-thin sectioning observations (Fig. 8P). Moreover, the nexine and intine were indistinguishable in the *atsec23ad* microspores (Fig. 9L). The inner structure of *atsec23a* and *atsec23d* microspores was comparable with that of the wild type (Fig. 9J, K). In contrast, most cellular inclusions of *atsec23ad* microspores degenerated and only a few distinguishable organelles remained (e.g. plastids and mitochondria) (Fig. 9L). Interestingly, the plastids in the incompletely degenerated *atsec23ad* pollen grains showed profound alteration in their morphologies compared with those of the wild type, *atsec23a*, and *atsec23d* (Fig. 9L; arrows). At the tricellular stage, mature pollen walls were completed by deposition of pollen coat materials in the wild-type, *atsec23a*, and *atsec23d* pollen grains (Fig. 9M–O). At this stage, wild-type and *atsec23d* pollen grains developed a thin uniformly distributed intine around the plasma membrane, while this layer was thicker and less electron-dense in *atsec23a* pollen grains (Fig. 9M–O; Supplementary Fig. S9A–C). Most pollen grains in the *atsec23ad* anthers degenerated, and flattened pollen shells, remnants of tapetum, and dead pollen materials were left inside the locules (Fig. 9P). Anthers of *atsec23ad* also contained few undegenerated pollen grains with incomplete pollen walls. Some of them had walls composed of sporopollenin-like aggregations with little pollen coat materials which contained numerous rod-shaped electron-lucent structures (Fig. 9P), while others had walls consisting of elongated bacula that were not covered by tectum and with many depositions of pollen coat materials (Supplementary Fig. S9D, E). These depositions were less electron dense and less compact than those of the wild type, *atsec23a*, and *atsec23d*, and contained many electron-lucent vesicle-like structures of different sizes. This suggested incomplete pollen coat depositions and a possible defect in the development of tapetal cells, the main source of pollen coat materials. These results indicated that the depletion of both *AtSEC23A* and *AtSEC23D* results in defective pollen wall development and consequently leads to pollen degeneration.

### Loss of *AtSEC23A* and *AtSEC23D* causes abnormalities in tapetum development

**Fig. 10.**
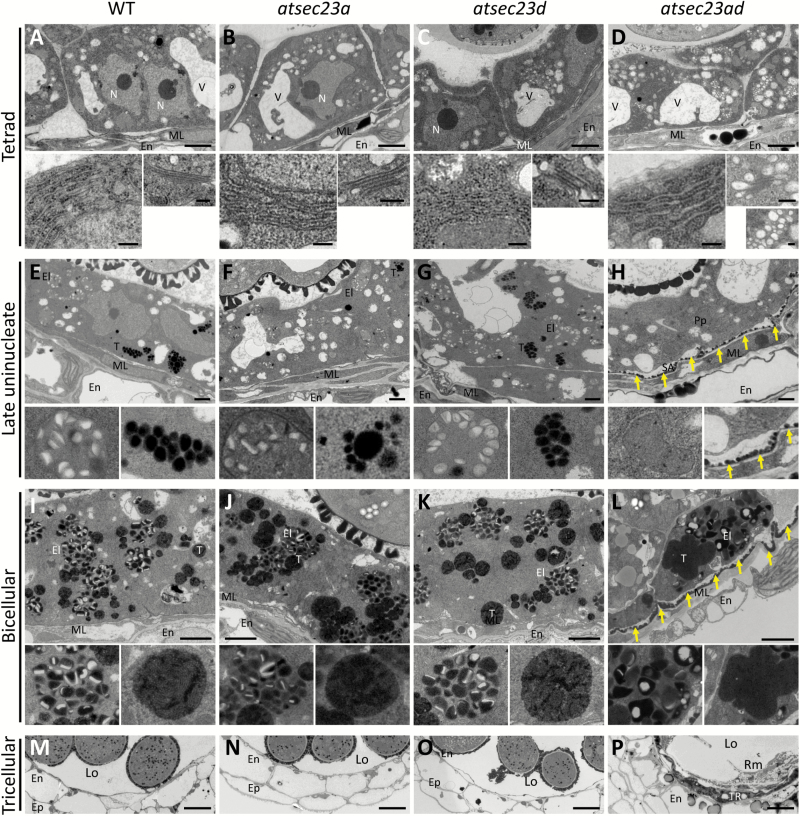
TEM micrographs of tapetal cells and their organelles in WT, *atsec23a*, *atsec23d*, and *atsec23ad*. (A–D) Ultrastructure of tapetal cells at the tetrad stage. The left side of the lower panels in (A–D) shows magnified images of the ER. The right side of the lower panels indicates magnified images of the Golgi in (A–C) or Golgi (top) and clusters of the tiny vesicles (bottom) in (D). (E–H) Ultrastructure of tapetal cells at the late uninucleate stage. Left and right sides of the lower panels in (E–G) show magnified images of the labeled elaioplasts and tapetosomes in the upper panels, respectively. Left and right sides of the lower panels in (H) indicate magnified images of the labeled proplastid (precursor of elaioplast) and sporopollenin-like aggregations on the middle layer that faces the tapetum in the upper panels, respectively. (I–L) Ultrastructure of tapetal cells at the bicellular stage. Left and right sides of the lower panels show magnified images of the labeled elaioplasts and tapetosomes in the upper panels, respectively. (M–P) Ultrastructure of anthers at the tricellular stage. Arrows indicate the sporopollenin-like aggregations on the locule wall. El, elaioplast; En, endodermis; Ep, epidermis; Lo, locule; ML, middle layer; N, nucleus; Pp, proplastid; Rm, remnants of pollen materials; SA, sporopollenin-like aggregations; T, tapetosome; Ta, tapetum; TR, tapetum residue; V, vacuole. Scale bars=2 µm (A–D and I–L; upper panels), 250 nm (A–D; lower panels), 1 µm (E–H), and 10 µm (M–P).

The sporophytic effect on pollen phenotype, apparent defects in microspores at the time of formation of significant exine walls, and the incomplete pollen coat in *atsec23ad* suggested the possibility of dysfunction of tapetal cells. Thus, we performed TEM observation focused on the endomembrane system and organelles in tapetal cells during different developmental stages. At the tetrad stage, tapetal cells in the wild type, *atsec23a*, and *atsec23d* showed several small and few large vacuoles, and contained elongated stacks of rough ER with clear ribosomes and well-defined Golgi bodies (Fig. 10A–C). In contrast, tapetal cells of *atsec23ad* had smaller vacuoles and clusters of tiny vesicles, and contained rough ER surrounded by less clear ribosomes and abnormal Golgi stacks swollen at their ends and connected with numerous irregular vesicles (Fig. 10D). The tapetum at the late uninucleate stage is characterized by the formation of two specialized storage organelles, the elaioplast and tapetosome. Well-developed elaioplasts with numerous electron-lucent plastoglobules and clusters of small electron-dense tapetosomes were evident in tapetal cells of the wild type, *atsec23a*, and *atsec23d* (Fig. 10E–G). In contrast, in *atsec23ad* tapetal cells, undifferentiated proplastids (precursors of elaioplasts) were observed and the formation of tapetosomes was not yet initiated (Fig. 10H), which indicated a delay in tapetum development. In addition, several sporopollenin-like aggregations were deposited on the middle layer that faces the tapetum (Fig. 10H; arrows), coinciding with the clear defects in microspore wall development (Fig. 9H). At the bicellular stage in the wild type, *atsec23a*, and *atsec23d*, tapetal cells continued to develop more abundant mature elaioplasts with definite membranes and fully developed tapetosomes (Fig. 10I–K). The tapetosomes became larger and contained numerous fragmented electron-dense structures. In contrast, the *atsec23ad* tapetal cells had elaioplasts without definite membranes consisting of fewer and larger plastoglobules (Fig. 10L). In addition, the tapetosomes lost their integrity and had unclear fragmented inner structures. Moreover, deposited sporopollenin-like aggregations on the middle layer became more abundant (Fig. 10L; arrows). At the tricellular stage, tapetal cells completely degenerated in the wild type, *atsec23a*, and *atsec23d* (Fig. 10M–O). However, the remnants of tapetal cells were still observed in *atsec23ad* anthers (Fig. 10P), indicating a delay in tapetum degeneration. These findings suggested that lack of *AtSEC23A* and *AtSEC23D* causes structural abnormalities and an incomplete development of tapetal cells.

## Discussion

The involvement of SEC23 proteins in regulating plant growth and development, and the functional differences among them have remained unknown. In the present study, we revealed that out of the seven *A. thaliana SEC23* homologs, *AtSEC23A* and *AtSEC23D* have divergent primary structures from that of other *AtSEC23* genes and play essential roles in pollen wall formation, exine patterning, and tapetum development. We also provided evidence for their partially different functions in intine formation and pollen germination.

Knockout of *AtSEC23D* caused a defect in the microspore exine but did not affect the intine (Fig. 9). Pollen grains of *atsec23d* exhibited less sporopollenin deposition, incomplete tectum formation, and disrupted reticulate architecture (Fig. 2). In contrast, knockdown of *AtSEC23A* caused defects in both exine and intine (Fig. 9). The exine of *atsec23a* pollen grains was also lacking the reticulate architecture and had wide areas with incomplete tectum formation and less sporopollenin deposition (Fig. 2), whereas the intine was thicker and less electron dense (Fig. 9; Supplementary Fig. S9). Moreover, pollen grains of *atsec23a* showed an impaired germination rate (Fig. 4J, N). Despite the low pollen germination rate, the *atsec23a* mutant was normally fertile, which may be due to the sufficient amount of functional pollen grains required for fertilization. Mutations in *AtSEC24A*, *AtSEC24B*, and *AtSEC31B*, other members of COPII, have also been shown to cause compromised pollen germination (Conger *et al.*, 2011; Tanaka *et al.*, 2013; Zhao *et al.*, 2016). During pollen germination, the growing pollen tube is encased only by the intine (Chebli *et al.*, 2012). Possibly, the thickened intine in *atsec23a* is responsible for the impaired pollen germination rate by increasing the required pressure for pollen germination. In *Brassica campestris*, knockdown of *BcMF8* (putative arabinogalactan protein gene) and double knockdown of *BcMF26a* and *BcMF26b* (polygalacturonase genes) causes reduced pollen germination and retarded pollen tube growth as a result of remarkable thickening of the intine (Lin *et al.*, 2014; Lyu *et al.*, 2015). In *atsec23a*, AtSEC23D had the main function in pollen development. However, since AtSEC23D lacks the β-barrel and gelsolin-like domains, it may not be able to complement some of the functions of AtSEC23A including the ER export of such proteins as BcMF8 and BcMF26s, explaining the compromised pollen germination in *atsec23a*.

The frequent microspore degeneration and leakage observed in *atsec23ad* locules were linked with loss of the intact pollen wall. Pollen wall integrity must be maintained to ensure normal pollen development. Mutations in genes that affect pollen wall integrity, such as *MALE STERILITY1* (*MS1*), *MS2*, *CALLOSE SYNTHASE5* (*CALS5*), *CYTOCHROME P450* (*CYP703A2*), *RUPTURED POLLEN GRAIN1* (*RPG1*), *DEFECTIVE IN EXINE PATTERNING1* (*DEX1*), *NO EXINE FORMATION1* (*NEF1*), *TRANSIENT DEFECTIVE EXINE* (*TDE1*), *ATP-binding cassette transporter G26* (*ABCG26*), and the COPII component *AtSEC31B*, have resulted in collapsed pollen grains with degenerated cytoplasm (Aarts *et al.*, 1997; Paxson-Sowders *et al.*, 1997; Wilson *et al.*, 2001; Ariizumi *et al.*, 2004, 2008; Dong *et al.*, 2005; Morant *et al.*, 2007; Guan *et al.*, 2008; Choi *et al.*, 2011; Paxson-Sowders *et al.*, 2001; Zhao *et al.*, 2016). Interestingly, the mutants *dex1*, *nef1*, *tde1*, *abcg26*, and *atsec31b* have accumulated sporopollenin-like aggregations on the middle layer similar to the *atsec23ad* mutant. These sporopollenin-like aggregations indicate an unsuccessful sporopollenin deposition and polymerization onto the microspore plasma membrane. One explanation is that the sporopollenin produced by tapetal cells in *atsec23ad* lacked components required for correct deposition and polymerization. In the *ms1* mutant, the collapsed microspores that tended to stick together were thought to be a result of an unusual chemical composition of pollen wall materials (Vizcay-Barrena and Wilson, 2006). The tendency of *atsec23ad* microspores to aggregate and stick together may support the above-mentioned explanation. Another possibility is that *AtSEC23A* and *AtSEC23D* may mediate transport of signaling proteins necessary for the co-ordination of sporopollenin deposition and polymerization onto the microspore plasma membrane. It was suggested that *ABCG26* may transport some signaling molecules required for the co-ordination of exine formation and sporopollenin polymerization on the developing microspore wall (Quilichini *et al.*, 2010). The study of the *tde1* mutant has suggested the existence of factors controlling the exine patterning and being potentially active at the uninucleate microspore stage (Ariizumi *et al.*, 2008).

Pollen wall development relies greatly on tapetum development. TEM analysis has revealed the occurrence of morphological abnormalities in organelles of tapetal cells (e.g. ER, Golgi, elaioplasts, and tapetosomes), and a delay in tapetum development and degeneration (Fig. 10). The endomembrane system in *atsec23ad* tapetal cells was highly affected. Lack of *AtSEC23A* and *AtSEC23D* affects the efficiency of COPII assembly, which may ultimately cause aberrant ER and Golgi structures and lead to the appearance of clusters of the tiny vesicles. Several studies have shown that maintaining the integrity of the ER and Golgi apparatus is associated with successful assembly of COPII proteins. For example, a mutation of SAR1 inhibits ER to Golgi trafficking and affects the Golgi integrity (Takeuchi *et al.*, 2000; Ward *et al.*, 2001). A missense mutation in *AtSEC24A* has resulted in defective ER and Golgi structures and has led to the formation of different sized vesicular clusters (Faso *et al.*, 2009).

In addition to the defective ER and Golgi, elaioplasts and tapetosomes in the *atsec23ad* tapetal cells exhibited a delayed development and structural abnormalities (Fig. 10). Tapetosomes originate from the ER and are enriched with triacylglycerols, flavonoids, and oleosins (Huang *et al.*, 2013), while elaioplasts develop from proplastids and are mainly filled with steryl esters and polar lipids (Piffanelli *et al.*, 1998; Wu *et al.*, 1999). It was suggested that the ER and plastids may exchange lipids at contact sites of their membrane (Andersson *et al.*, 2007; Fan *et al.*, 2015; Li *et al.*, 2016). The structural abnormalities and the delayed development of elaioplasts and tapetosomes may be a consequence of defective ER structures or a defective lipid exchange between the ER and plastids. Another possibility is that lack of *AtSEC23A* and *AtSEC23D* causes less efficient COPII assembly at the ERESs and disrupts transport of unidentified components required for normal elaioplast and tapetosome development. Knockout lines of *AtSEC31B* also showed a retarded development of elaioplasts and tapetosomes, with ultrastructural abnormalities (Zhao *et al.*, 2016). However, the mechanisms by which COPII components affect the formation of elaioplasts and tapetosomes require further investigation.

The *atsec23ad* mutant showed abnormal pollen coat depositions which were less electron dense and less compact than those of the wild type and abnormally contained many electron-lucent vesicle-like and rod-shaped structures (Fig. 9P; Supplementary Fig. S9). Similar abnormal depositions were previously observed in *atsec31b* and *abcg9 abcg31* mutants (Choi *et al.*, 2014; Zhao *et al.*, 2016). These abnormal depositions may be a direct result of the abnormal development of elaioplasts and tapetosomes in tapetal cells and/or the delay in tapetum degeneration. Alternatively, it may be due to less efficient ER export of proteins that participate in pollen coat formation. Recently, it was shown that ABCG9, which is involved in the transport of lipidic components required for pollen coat formation (Choi *et al.*, 2014), is probably transported through the ER–Golgi route (Zhao *et al.*, 2016).

In summary, our results showed that *AtSEC23A* and *AtSEC23D* are required for proper pollen wall development and exine patterning possibly by mediating efficient ER export of some essential proteins/enzymes participating in pollen wall formation. Our work provides a direct link between the early secretory pathway in tapetal cells and sporopollenin deposition/exine patterning. Moreover, our results indicated that AtSEC23A and AtSEC23D may not fulfill exactly the same functions; *AtSEC23D* functions only sporophytically in the tapetum to participate in exine formation, whereas *AtSEC23A* redundantly functions with *AtSEC23D* in exine formation, and additionally is involved in intine formation and pollen germination. Thus, we propose that *AtSEC23A* and *AtSEC23D* may have differing substrate preferences, providing evidence of functional diversity of SEC23 proteins in *A. thaliana*. Identifying the specific substrate and the interaction partners of *AtSEC23A* and *AtSEC23D* will be the basis for a future study.

## Supplementary data

Supplementary data are available at *JXB* online.

Table S1. Oligonucleotides used in this study.

Fig. S1. Multiple sequence alignment of SEC23 family proteins in yeast and *A. thaliana*.

Fig. S2. Pollen morphology in T-DNA insertion lines for the remaining *AtSEC23* genes and in complemented *atsec23a* and *atsec23d* lines.

Fig. S3. Normal functionality of the female gametophyte of *atsec23ad*.

Fig. S4. Phenotypes in the double mutant *atsec23ad*.

Fig. S5. Sporophytic control of *AtSEC23A* and *AtSEC23D*.

Fig. S6. Quantitative co-localization analyses of AtSEC23A and AtSEC23D.

Fig. S7 Co-localization analyses of AtSEC23A and AtSEC23D in *N. benthamiana* leaf epidermal cells.

Fig. S8. Aniline blue staining of microspores at the tetrad stage.

Fig. S9. Abnormal thickening of the intine of *atsec23a* pollen grains and defective wall development in *atsec23ad* pollen grains.

Movie S1. Time-lapse confocal imaging of *N. benthamiana* leaf epidermal cells co-expressing AtSEC23A–G3GFP and the ERES marker AtSEC24A–TagRFP.

Movie S2. Time-lapse confocal imaging of *N. benthamiana* leaf epidermal cells co-expressing AtSEC23D–G3GFP and the ERES marker AtSEC24A–TagRFP.

Supplementary Table_Figures

Supplementary Movie S1

Supplementary Movie S2

## Abbreviations:

ABRC: Arabidopsis Biological Resource Center
CLSM: confocal laser scanning microscope
COPII: coat protein complex II
ER: endoplasmic reticulum
ERES: ER exit site
GUS: β-glucuronidase.
