# Supplementary material for: The Arabidopsis COPII components, AtSEC23A and AtSEC23D, are essential for pollen wall development and exine patterning
Source: J Exp Bot. 2018 Jan 30;69(7):1615–33. doi: 10.1093/jxb/ery015 (PMC5889017; doi:10.1093/jxb/ery015)
Supplement: Supplementary Table_Figures [file ery015_suppl_supplementary_table_s1_figures.pdf]

Table S1. Oligonucleotides used in this study.

Oligos	Sequence*
*att*B1 adaptor	5'-GGGGACAAGTTTGTACAAAAAAGCAGGCT-3'
*att*B2 adaptor	5'-GGGGACCACTTTGTACAAGAAAGCTGGGT-3'
P_{nos}-*att*B4	5'-GGGGACAACTTTGTATAGAAAAGTTGGCTGAGACACTATCATGAGCGGAGAATTAAGG-3'
P_{nos}- *att*B1r	5'-GGGGACTGCTTTTTTGTACAAACTTGTGACACTAGATCCGGTGCAGATTATTTG-3'
$P_{AtSEC23A}$-*att*B1	5'-GGGGACAAGTTTGTACAAAAAAGCAGGCTCCGACACTCGGGATTATTGATGGGAAATC-3'
$P_{AtSEC23A}$-*att*B2	5'-GGGGACCACTTTGTACAAGAAAGCTGGGTAGACACTCGGATTCCGAAGTTTCTACTT-3'
$P_{AtSEC23A}$-*att*B4	5'-GGGGACAACTTTGTATAGAAAAGTTGGCTGAGACACTCGGGATTATTGATGGGAAATC-3'
$P_{AtSEC23A}$-*att*B1r	5'-GGGGACTGCTTTTTTGTACAAACTTGTGACACTCGGATTCCGAAGTTTCTACTT-3'
$P_{AtSEC23D}$-*att*B1	5'-GGGGACAAGTTTGTACAAAAAAGCAGGCTCCGACACTCTGGAAAACCTATTCAAGCCA-3'
$P_{AtSEC23D}$-*att*B2	5'-GGGGACCACTTTGTACAAGAAAGCTGGGTAGACACTTGTTTCAGATCAGATCCTTCC-3'
$P_{AtSEC23D}$-*att*B4	5'-GGGGACAACTTTGTATAGAAAAGTTGGCTGAGACACTCTGGAAAACCTATTCAAGCCA-3'
$P_{AtSEC23D}$-*att*B1r	5'-GGGGACTGCTTTTTTGTACAAACTTGTGACACTTGTTTCAGATCAGATCCTTCC-3'
AtSEC23A-*att*B1	5'-AAAAAGCAGGCTCCGACACTATGGCTAACTTACCGAAATC-3'
AtSEC23A-*att*B2	5'-AGAAAGCTGGGTAGACACTCCTGGGCTCAGGAGGCAC-3'
AtSEC23D-*att*B1	5'-AAAAAGCAGGCTCCGACACTATGGCAGTGAGAGCAACGGT-3'
AtSEC23D-*att*B2	5'-AGAAAGCTGGGTAGACACTCTTCATGTATTCAGTGACAC-3'
RT-*AtSEC23A*-F	5'-CACATTCAGAAACTCACGAG-3'
RT-*AtSEC23A*-R	5'-CCTGGGCTCAGGAGGCAC-3'
RT-*AtSEC23D*-F	5'-GCCTCTCTGGAAGATGGAGT-3'
RT-*AtSEC23D*-R	5'-CTTCATGTATTCAGTGACAC-3'
ACT2-F	5'-CATCTTCTTCCGCTCTTTCTTTCCA-3'
ACT2-R	5'-CTCTTACAATTTCCCGCTCTGCTGT-3'
GN-*AtSEC23A*-F	5’-CATAAGGTGAGTCTGCAGCT-3'
GN-*AtSEC23A*-R	5’-GACGTCGGTTAATACCACGT-3'
GN-*AtSEC23D*-R	5’-GAGATTAGCTTGTAAGCTTG-3'
T-DNA-LB	5’-GCAATCAGCTGTTGCCCGTCTCACTGGAG-3'

*Solid and dotted underlines indicate regions corresponding to full-length *att*B sequences (*att*B1 and *att*B2; 25 bp, *att*B4 and *att*B1r; 22 bp), and regions corresponding to partial *att*B1 and *att*B2 sequences (12 bp), respectively.

```
ScSEC23    ------------------------------------------------------MDFETN--------EDINGVR-------------------------FTWNVFPST-----------  22
AtSEC23A   MANLPKSSVNYPGTLTPLEPNRPSPQPDRTPVPHSPPVVASPIPPRFPQPSFRPDQMSSPSMKSPSLLSPANGIRTGSPIPRLSTPPGPPVFNTPVKPAAVPFRTSPATPQPMAYSSANS  120
AtSEC23B   -----------------------------------------------------MSEMAS------MDPEGIDGVR-------------------------MTWNVWPRT-----------  25
AtSEC23C   -----------------------------------------------------MAEFGE--------LEAQDGVR-------------------------MPWNIIPVATK---------  25
AtSEC23D   -----------------------------------------------------------------------MAVR-------------------------ATVSRFPID-----------  13
AtSEC23E   -----------------------------------------------------MAETAN------TDLEGIDGVR-------------------------MTWNVWPHS-----------  25
AtSEC23F   -----------------------------------------------------MAEMADKAKVEEMDWEGIDGVR-------------------------MTWNLWPRT-----------  31
AtSEC23G   ------------------------------------------------------MDFLE--------LEAIEGLR-------------------------WSWNSWPTT-----------  22

ScSEC23    -------------------------------------------------RSDANSNVVPVGCLYTPLK----EYDELNVAPYNPVVCSGPHCKSILNPYCVIDPRNSSWSCPICNSRNHL  89
AtSEC23A   SLPVSTPSFYSNGSSVGSQRDLPDVVRMEEPIAADSPYVLFSANKVLKQKKLANVASLGFGAIVSAGRE---ISPGPQIIQRDPHRCLN--CGAYSNPYSSILIGSGQWQCVICENMNGS  235
AtSEC23B   -------------------------------------------------KVEASKCVIPVAACISPIR----YHRDIPSVEYAPLRCR--ICTAALNPFARVDFLAKIWICPICFQRNHF  90
AtSEC23C   -------------------------------------------------KEQSIDSEVPVSAIYTPLKP---LRSQSLLLPYSPLRCR--TCRSVLNPYSVVDFSACNWGCPFCFNRNPF  91
AtSEC23D   -------------------------------------------------SDAQEASGLPWGLTVTPFAAKDENGIGPACGSNGHLLPRCENCYAYFNTYCELD--QWAWNCSLCGTLNGL  82
AtSEC23E   -------------------------------------------------KAEASKCVIPLAACISPIR----RHADIPTLPYAPLRCR--TCSAALNAYAQVDFTAKLWICPFCYQRNHF  90
AtSEC23F   -------------------------------------------------KVEASKCVIPLAASISPIR----RHPLILDLPYAPLDCK--TCKALLNAFARVDFAAMNWVCPFCYHRNHF  96
AtSEC23G   -------------------------------------------------KSDCESLVVPLSIMYTPLM----HFSELPTIPYDPLICS--RCGAVLNPYARVDYQSRIWSCPFCFHKNLF  87

ScSEC23    PPQYTN-LSQENMPLEL--QSTTIEYITNKP--------------------------VTVP----------------------PIFFFVVDLTSETENLDSLKESIITSLSLLPPNALIG  158
AtSEC23A   KGEYVA-SSKNELQNFPELSLPLVDYVQTGNKRPG----------------FVPASDSRTS----------------------APVVLVIDECLDEPHLQHLQSSLHAFVDSLPQTTRLG  316
AtSEC23B   PPHYHV-MSETNVPCELYPQYTTVEYTLPNPSQP---------TGVGNFDQTGAVSGQPSP----------------------SVFVFVLDTCMIEEEFGYAKSALKQAIGLLPENALVG  178
AtSEC23C   PLNYSS-VADNNLPPELFPHSTTVEYLCDS-------------------------FSSPSP----------------------PVFLFVVDTCLISEELDFLKSSLFQALDLLPDTSILG  163
AtSEC23D   PSDAIARYSNPHSIPEMTSSFIDLEMPLDGS----------------------EEEMTQAR----------------------PVYVAAIDISSSEEFLELTKSALLAALEALSPGALFG  158
AtSEC23E   PPHYHV-ISETNLPGELYPQYTTVEYTLPPP--------------------VANGEGLVDP----------------------PVFVFVLDTCMIEEELDFAKSALKQAIGLLPENALVG  167
AtSEC23F   PSHYHS-ISEINLPGELYPQYTTVEYTLPP-----------------------DPSRVPPP----------------------PVFVFVLDTCMIEEELGYAKSALKQAIGLLPENALVG  170
AtSEC23G   PRSYSG-ITETNLPAELFPTYSAVEYSPLPSRQSGSNTTTPTAAASWSNGFNQGVRSMPSNSSFSSLASSTVGGGGGVISELGPAFVFVVDASMVEDELRAVRSDVLFVIEQLPENCLVA  206

ScSEC23    LITYGNVVQLHDLS--SETIDRCNVFRGDREYQLEALTEMLTGQKPTGPGGAASHLPNAMN--KVTPFSLNRFFLPLEQVEFKLNQLLENLSPDQWSVPAGHRPL---------RATGSA  265
AtSEC23A   IILYGRTVSIYDFS--EDSVASADVISGAKSPSAESMKALIYGTG---------------------------VYLSPMHASLKVAHEIFSSLRPYTLNVPEASRD---------RCLGTA  398
AtSEC23B   FVSFGTQAHVHELG--FSDLTKVYVFRGDKEISKDQVLEQLGLGASGRRNPVGGFPMGRDNSANFGYSGVNRFLLPASDCEFTIDLLLEELQTDQWPVQAGRRQS---------RCTGVA  287
AtSEC23C   LITFDSLVRVYELG--FPHCTKSYFFHGNKDCTKDQLLDQLSFFVKNPK-PSSGVIAGARD--GLSSDDIARFLLPASDCHFTLHSVLEELGNSPWPVAADHRPA---------RCTGVA  269
AtSEC23D   LVTFSHKIGLYDVQGPIPVVKNVFIPPDGESSLSLELEDVMP---------------------------LLQFLAPVETCKDRIAAALETLRPITSWERSAGAGQGMDSVLMGGRGFGTA  251
AtSEC23E   FVSFGTQAHVHELG--FSEMSKVFVFKGDKEISKDQILDQLGLGGSSRR----GGSKGPQN--GFPSSGLNRFLLPASECEFTLNSLLDELQSDQWPVKPGHRSQ---------RCTGVA  270
AtSEC23F   FVSFGTQAHVHELG--FSEMSKVFVFKGNKEVTKDQILDQLGLGSSSRRAPTSGFSKGAQN--GFQSSGVDRFLLPASECEYTLDLLLDELQSDQWPVQPGHRPQ---------RCTGVA  277
AtSEC23G   LITFDSMVRVYDLG--FSECSKVVVFHGERDLSPDQIQQFLGLG----------YSKQFHHG-KMSAIRKQSFLLPLVECEFNLTSAFEEIIP-LVDVKPGHRPH---------RSTGAA  303

ScSEC23    LNIASLLLQGCY--KNIPARIILFASGPGTVAPGLIVNSELKDPLRSHHDIDSDHAQHYKKACKFYNQIAQRVAANGHTVDIFAGCYDQIGMSEMKQLTDSTGGVLLLTDAFSTAIFKQS  383
AtSEC23A   VEAALAIIQGPS--AEMSRGVVRRAGGNSRIIVCAGGPITYGPGSVPHSMSHPNYPYMEKTAIKWMENLGREAHRHNTVVDILCAGTCPLRVPILQPLAKASGGVLVLHDDFG-EAFGVD  515
AtSEC23B   ISVATGLLGACF--PGTGARIVALIGGPCSEGPGTIVSKDLSEPLRSHKDLDKDAAPFYKKAEKFYDALANQLVNQGHVLDLFASALDQVGVAEMKAAVERTGGLVVLSESFGHSVFKDS  405
AtSEC23C   LRIAASLLGACF--PGSAARIMAFIGGPSTQGPGAIVSRELSDPIRSHKDIDKDSAMYYHKAVEFYEMLAKQLVHQGHVLDVFASSVDQVGIAELKVAVEQTGGFVVLAESFGHSVFRDS  387
AtSEC23D   MEALFNYLGSEFGNTFALARVFAFLSGPPDYGRGQLDTSRYGEQYASKRVDADR--ALLPEQTPFYKDLATIAVQSGVCVDLFAVTNEYTDLASLKFLSIESGGSLFLYSSTDDSTLPQD  369
AtSEC23E   LSVAAGLLGACL--PGTGARIVALIGGPCTEGPGTIVSKDLSDPVRSHKDLDKDAAPYYKKAVKFYDSIAKQLVTQGHVLDLFASALDQVGVAEMKVAVERTGGLVVLSESFGHSVFKDS  388
AtSEC23F   LSVAAGLLGACL--PGTGARIVALVGGPCTEGPGTIISKDLSDPVRSHKDLDKDAAPYYKKAVKFYDSIAKQLVAQGHVLDLFASALDQVGVAEMKVAVESTGGLVVLSESFGHSVFKDS  395
AtSEC23G   ISTALGLLEGCS--VTTGSRIMVFTSGPATRGPGIIVDSDLSNSIRTHRDIITGHVSYYDKSCGFYKKLAKRLCDSSVVLDVFACSLDQVGAAELRYAVEMSGGFLLLGETFESEQFKKC  421

ScSEC23    YLRLFAKDEEGYLKMAFNGNMAVKTSKDLKVQGLIGHAS-AVKKTDANNISESEIGIGATSTWKMASLSPYHSYAIFFEIANTAANSNPMMSAPGSADRPHLAYTQFITTYQHSSGTNRI  502
AtSEC23A   LQRAAT------RAAGSHGLLEVRCSDDILITQVIGPGE-EAHSETHE-----TFKSDAALSIQMLSVEETQSFSLSME------NKRDIKSD--------HVFFQFAFHYSDVYQADVS  609
AtSEC23B   FKRVFE-DGEESLGLCFNGTLEICCSKDIKQGIIGPCA-SLQKKGPS-VADTVIGEGNTTQWKMCGLDKRTCLTVFFDLSSSDQSSAPGGVN-------NQLYLQFMTSYQNSKGKTLQ  515
AtSEC23C   LKRVCQ-SGENDLGLSSCGIFEINCSKDIKVQGIIGPCA-SLEKKGPL-CSDTAIGQGHTSAWKMCGLDNNTSICLVFEIAKIDTADVVLQSQS------NQFYFQFLTYYQHSNGQTRL  498
AtSEC23D   MFRMLN------RPYAFNCVLRLRTSTEFKPGNSFGHFFPDPQYENLQHIICCDSYATYAYDFEFADNTGFSRHSGEQPVVQIAFQYTVVVPP--------EGLSNSEMSSSSRGKHTLQ  475
AtSEC23E   FKRVFE-DGDQALGLCFNGTLEICCSQDIKIQGAIGPCS-SLEKKGAS-VADTVIGEGNTSAWRLCGLDKTTCLTIFFDISSSG-SNTPGAAN-------PQFYLQFLTSYQNPEGQTLL  497
AtSEC23F   FKRMFE-DGEHSLGLCFNGTLEINCSKDIKIQGVIGPCS-SLEKKGPN-VADTVIGEGNTSAWKLCGLDKSTCLTVFFDLSSTG-STAPGALN-------QQLYLQFITRYQNSEGKSLA  504
AtSEC23G   LRHIFIRDADGNLSMYFDVSLEVVTTKDMRICGALGPVV-SLRQKNDI-VSETEIGEGGTYMWKTSTVTNKTCVSFFFHVSNEQ-NRKPQPGS--------AFFIQFITRYRYGNGAMRK  530

ScSEC23    RVTTVANQLLPFGTP--AIAASFDQEAAAVLMARIAVHKAETD-DGADVIRWLDRTLIKLCQKYA-------------DYNKDDPQSFRLAPNFSLYPQFTYYLRRSQFLSVFNN--SPD  604
AtSEC23A   RVITFK---LPTVDSISAYLQSVEDEASAVLISKRTLLLAKNQKDAVDMRATVDERIKDIALKFG-------------SQVP-KSKLYSFPKELSSLPELLFHLRRGPLLGNIIG--HED  710
AtSEC23B   RVTTVTRQWVDTGLSTEELVQGFDQETAAVVVARLASLKMETE-EGFDATRWLDRNLIRLCSKFG-------------DYRKDDPASFTLNPNFSLFPQFTFNLRRSQFVQVFNN--SPD  619
AtSEC23C   RVTTLSRRWVMGTESLQELSNGFDQEAAAVVMARLISSKMETQ-PEFNPQRWVDKALINLCTWFG-------------DYQKGNPSSFSLSSQLSIFPQFVFHLRRSQFVQVFNN--SPD  602
AtSEC23D   RRLRIRTMQFGTAHNINEIYDSVDHEVVLSLLVHKVILASLED-GVREGRALLHDWLVILTAQYNDAFNLVQYKNGNKSMSSQIDITFSQCPQLEPLPRLVFALLRNPLLRFHEEGVHPD  594
AtSEC23E   RVTTVCRQWIDSAVSSEELVQGFDQETAAVVMARLASLKMESE-EGFDATRWLDRNLIRLCSKFG-------------DYRKDDPASFTLNPYFSLFPQFIFNLRRSQFVQVFNN--SPD  601
AtSEC23F   RVTTLTRQWVDTAVSTENLVQGFDQETAAVVMARLTSLKMETE-EGFDATRWLDRTLIRLCSKFG-------------EYRKDDPTSFTLKPYLTLFPQFMFNLRRSQFVQVFNN--SPD  608
AtSEC23G   RVTTVARRWVAGKSP--EISSSFDQETAASVMARLAINRAEEC-HARDVITWLDNGLIRFASRFG-------------DYIQEDPSSFRLTPNFSLYPQFMFYLRRSQFLDVFNN--SPD  632

ScSEC23    ETAFYRHIFTREDTTNSLIMIQPTLTSFSMEDDPQPVLLDSISVKPNTILLLDTFFFILIYHGEQIAQWRKAGYQDDPQYADFKALLEEPKLEAAELLVDRFPLPRFIDTEAGGSQARFL  724
AtSEC23A   ERSVLRNLFLNASFDLSLRMVAPRCLMHQEGGTFEELPAYDLSMQSDKAVILDHGTDVFIWLGAELS----------ADEVKSAAVLAACRTLAEELTEFRFPAPRILAFKEGSSQARYF  820
AtSEC23B   ETAYNRMLLNRENISNAAVMIQPSLTTYSFNSLPQPALLDVASIGADRILLLDSYISVVVFHGMTIAQWRNLGYQNQPEHQAFAQLLEAPQEDAQMIIRDRFPVPRLVVCDQHGSQARFL  739
AtSEC23C   ETAYFRMILYRENVSNSVVMIQPSLISFSFHSPPEPILLDVASIAADRILLLDSYFTLVIFHGSTIAQWRKAGYHNQPEHQAFGHLLQSPRDYADTIMSERFPTPRLVICDQYGSQARFL  722
AtSEC23D   YRIYLQCLFSVLDPSSLHCGIYPALMSYS---TPDTLAYPRHSLSRAALITSGSPIFFLDAYTTLIVFYSSTADPSIPFPPPQDCLLR---QTINKVKQERSITPKLVFIRGGRDDATVF  708
AtSEC23E   ETAYFCMLLNRENISNATVMIQPSLTTYSFNSPAEPALLDVASIAADRILLLDAYFSVVVFHGMTIAQWRNMGYHHQPEHQAFAELLQAPQEDSQMIVRERFPVPRLVVCDQHGSQARFL  721
AtSEC23F   ETAYFRMLLNRENISNAIVMIQPSLTSYSFNSGPQAALLDVASIAADKILLLDAYFSVVVFHGMTISQWRNMGYHHQPEHEAFAQLLQAPQEDSQMLVRERFPVPRLVVCDQHGSQARFL  728
AtSEC23G   ETGFFRLMLNREGVVNSIIMIQPTLLRYSFDGPPVPVLLDIRSVTPDVILLFDSYFYVVIHHGSKIAQWRKLEYHKDPSHETFRNLLEAPEIDAAQLVTDRIPMPRIVRCDQHGSQARFL  752

ScSEC23    LSKLNPS-----------------DNYQDMARGGSTIVLTDDVSLQNFMTHLQQVAVSGQA  768
AtSEC23A   VCRLIPAHKDPPYEQEARFPQIRTLTTEQRMKLKSSFIEFDEASFCEWMRSLKVVPPEPR-  880
AtSEC23B   LAKLNPSA---------------TYNNASEMNAGSDIIFTDDVSLQVFFQHLQKLAVQS--  783
AtSEC23C   LAKLNP-------------------CDGDAHFSGQSNVFTDDVSLSVFLDHLRRLIVH---  761
AtSEC23D   ENYLIEEQ---------------DVDGNGFASAMGFVSFLDDISQRVTEYMK---------  745
AtSEC23E   LAKLNPSA---------------TYNNANEMSTGSDVIFTDDVSLQVFFEHLQKLVVQS--  765
AtSEC23F   LAKLNPSA---------------TYNNANEMSAGSDIIFTDDVSLQVFIEHLQKLAVQS--  772
AtSEC23G   LAKLNPS-----------------VTQKTDHTGGSDIVLTDDMSLQDFLEDLQSLAVKG--  794
```

Supplementary Fig. S1. Multiple sequence alignment of SEC23 family proteins in yeast and *A. thaliana*. The yeast and *A. thaliana* amino acid sequences identical to those used in Fig. 1A were re-aligned by the ClustalW ver. 1.83 program (http://clustalw.ddbj.nig.ac.jp/). The five domains shown in Fig. 1 are indicated by colored letters: zinc finger (orange), trunk (green), β-barrel (blue), all-helical (red), and gelsolin-like (purple). The previously-reported conserved amino acid residues are highlighted by yellow.

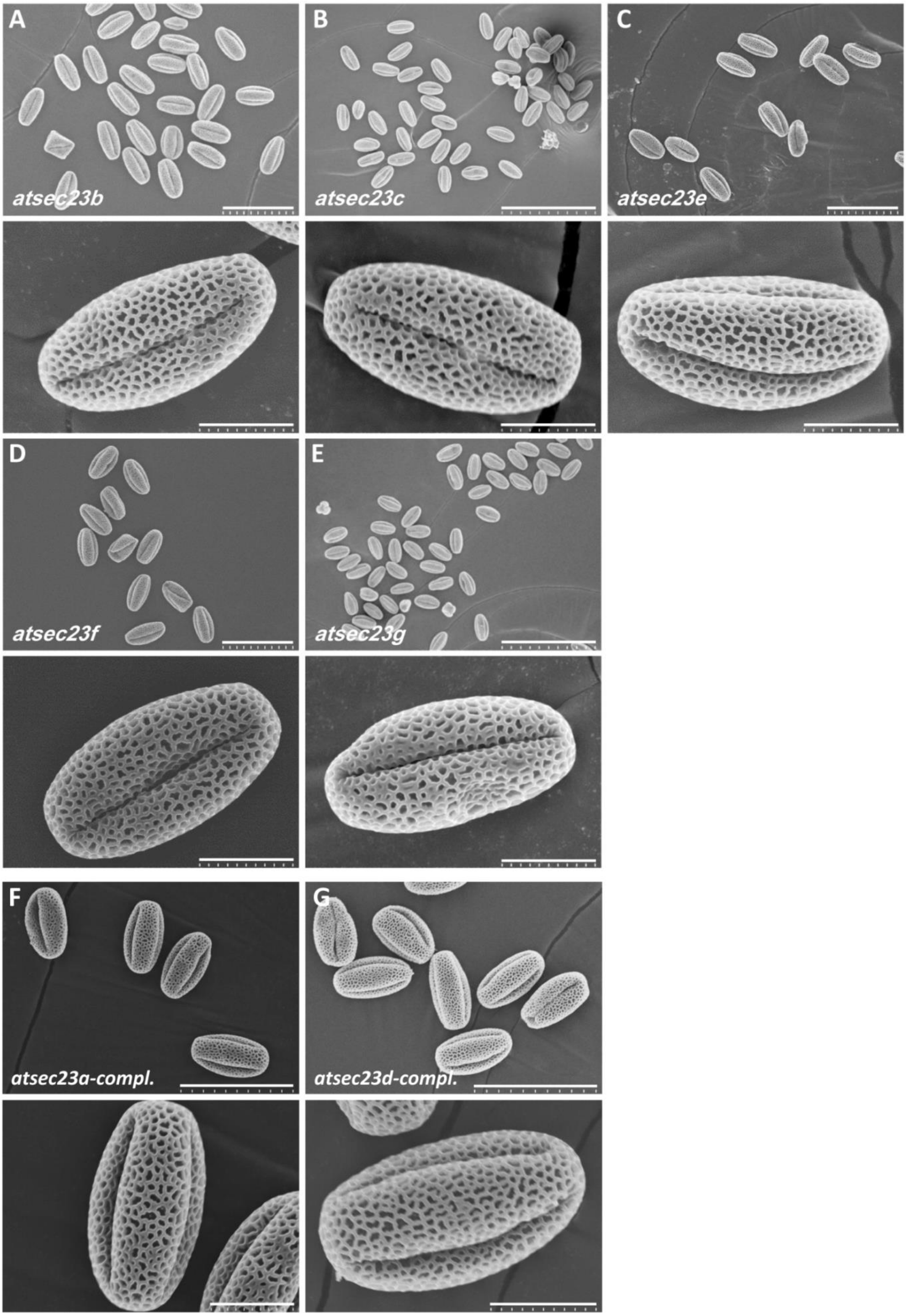
A
atsec23b
B
atsec23c
C
atsec23e
D
atsec23f
E
atsec23g
F
atsec23a-compl.
G
atsec23d-compl.

Supplementary Fig. S2. Pollen morphology in T-DNA insertion lines for the remaining *AtSEC23s* and in complemented *atsec23a* and *atsec23d* lines. (A-E) SEM micrographs of pollen grains in T-DNA insertion lines for the *AtSEC23B*, *AtSEC23C*, *AtSEC23E*, *AtSEC23F* and *AtSEC23G*. The lines, SALK_051290 (*atsec23b*), SALK_075252 (*atsec23c*), SALK_080595 (*atsec23e*), SALK_104305 (*atsec23f*), and SALK_027036 (*atsec23g*) were used. (F-G) SEM micrographs of pollen grains in complemented *atsec23a* (F) and *atsec23d* (G) lines. Scale bars = 50 μm in (A, C, D, F and G; upper panels), 100 μm in (B, E; upper panels), and 10 μm in the lower panels.

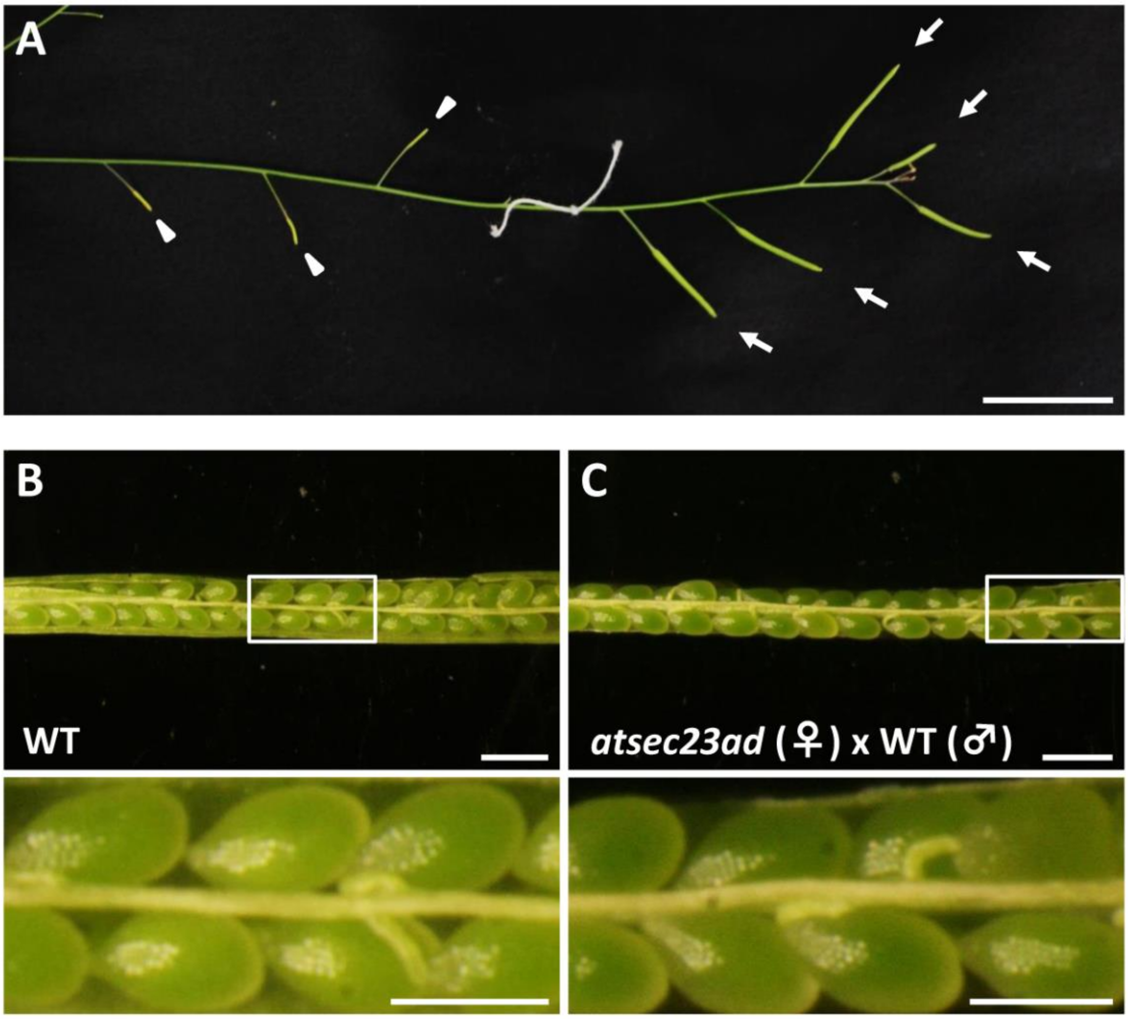

Supplementary Fig. S3. Normal functionality of the female gametophyte of *atsec23ad*. (A) An *atsec23ad* branch with normal elongated siliques after pollination with WT pollen grains. Arrows and arrowheads indicate crossed and self-pollinated siliques, respectively. (B, C) Seed development in siliques of WT and *atsec23ad* pollinated by WT pollen grains. Lower panels are magnifications of the boxed areas in upper panels. Scale bars = 2 cm in (A), 1 mm in (B, C; upper panels), and 0.5 mm in (B, C; lower panels).

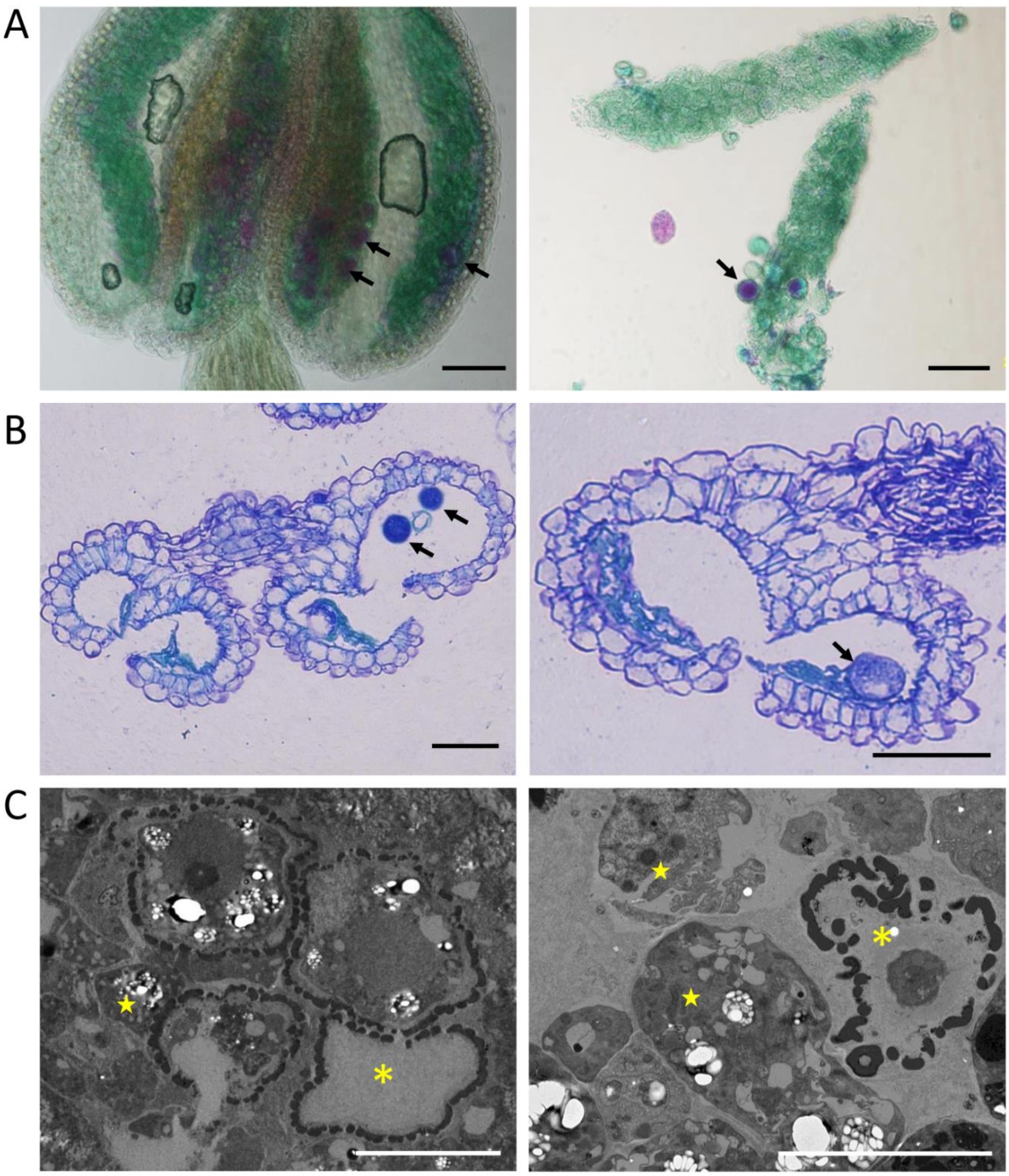

Supplementary Fig. S4. Phenotypes in the *atsec23ad* double mutant. (A) Alexander's staining of *atsec23ad* anthers showing a few positively stained pollen grains (arrows). (B) Technovit semi-thin sections of *atsec23ad* anthers showing a few intact pollen grains indicated by arrows. (C) TEM micrographs of *atsec23ad* microspores at the bicellular stage showing the severe defects. Stars and asterisks show naked microspores with no walls and empty walls without microspore cells, respectively. Scale bars = 50 μm in (A, B) and 10 μm in (C).

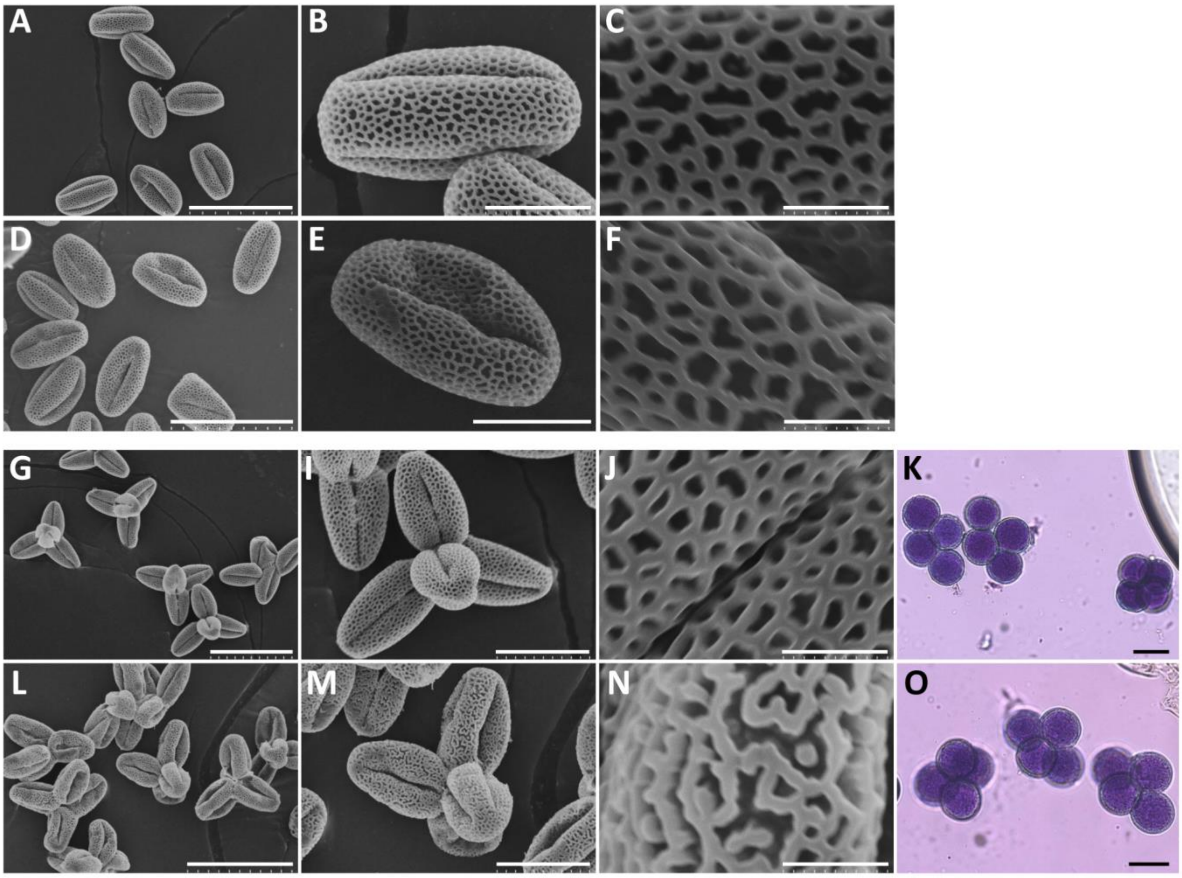

Supplementary Fig. S5. Sporophytic control of *AtSEC23A* and *AtSEC23D*. (A-F) SEM micrographs of pollen grains and their surface structure in heterozygous lines of *atsec23a* (+/*atsec23a*) (A-C) and *atsec23d* (+/*atsec23d*) (D-F). (G-O) Tetrad analysis of heterozygous lines of *atsec23a* in the background of *atsec23d* and *qrt1-2*. SEM micrographs of pollen-tetrads of *qrt1-2/qrt1-2* (G-J) and heterozygous line of *atsec23a* in the *atsec23d* and *qrt1-2* background (+/*atsec23a*, *atsec23d/atsec23d*, *qrt1-2/qrt1-2*) (L-N). Alexander's staining of pollen-tetrads of *qrt1-2/qrt1-2* (K) and +/*atsec23a*, *atsec23d/atsec23d*, *qrt1-2/qrt1-2* (O). Scale bars = 50 µm in (A, D, G, and L), 10 µm in (B, E, I, and M), 3 µm in (C, F, J, and N), and 20 µm in (K and O).

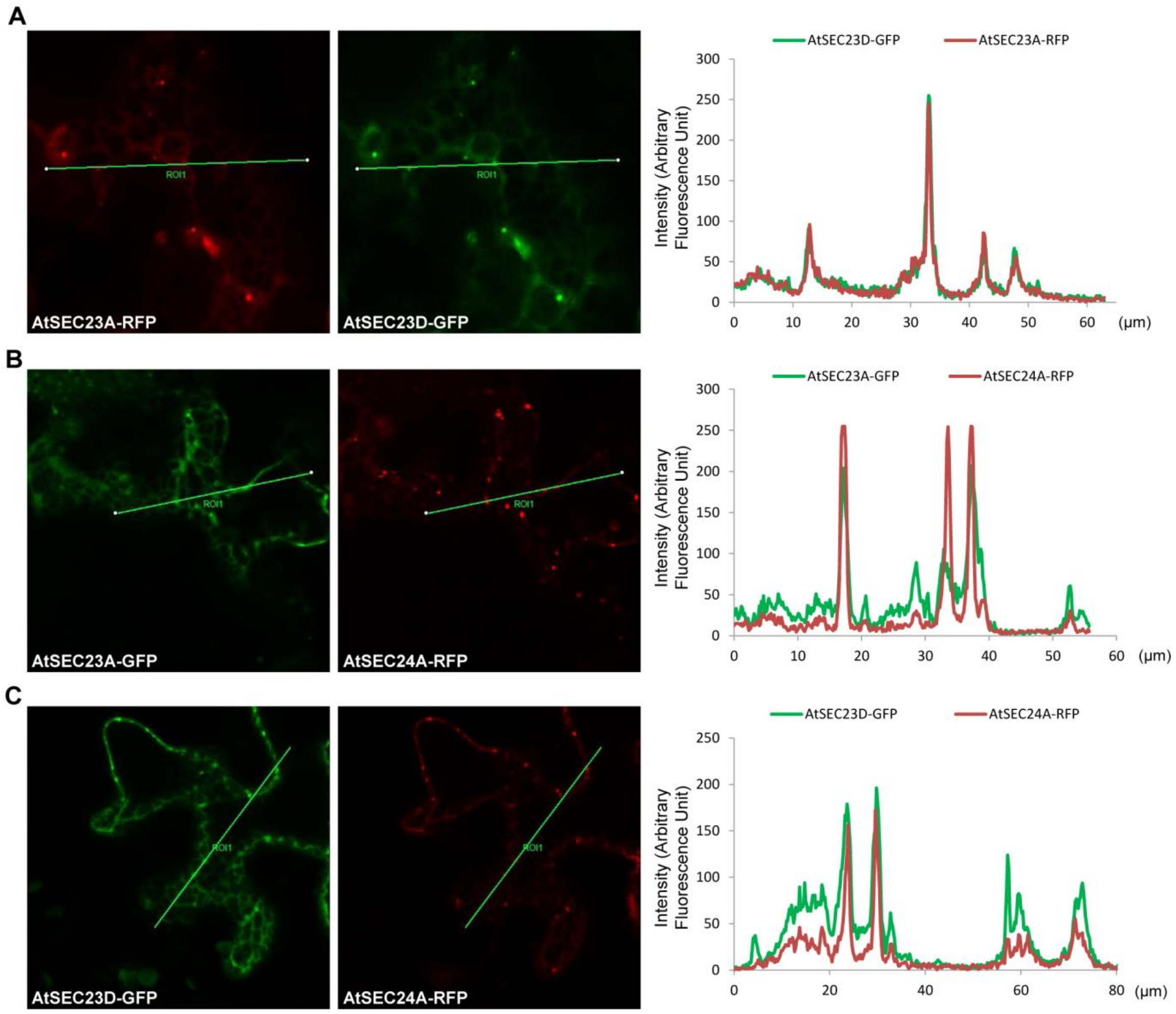

Supplementary Fig. S6. Quantitative co-localization analyses of AtSEC23A and AtSEC23D. (A) Fluorescent images of an epidermal cell co-expressing AtSEC23D-G3GFP and AtSEC23A-TagRFP. (B, C) Fluorescent images of epidermal cells co-expressing AtSEC23A-G3GFP (B) or AtSEC23D-G3GFP (C) with the ERES marker AtSEC24A-TagRFP. Fluorescence intensity on regions of interest (ROIs) indicated in each image was quantified by the software, LAS AF (Leica Microsystems, Wetzlar, Germany) and was plotted on right graphs.

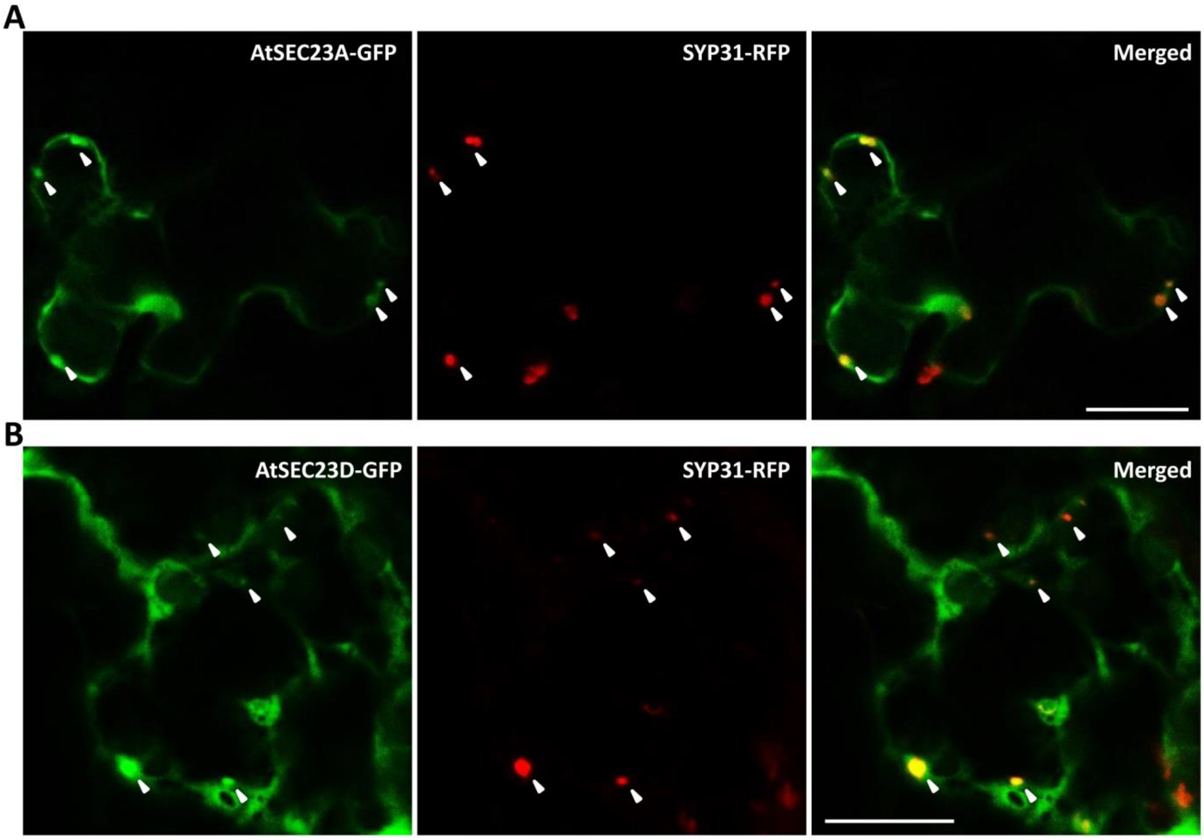

Supplementary Fig. S7. Co-localization analyses of AtSEC23A and AtSEC23D in *N. benthamiana* leaf epidermal cells. Confocal images of *N. benthamiana* leaf epidermal cells co-expressing AtSEC23A-G3GFP (A) or AtSEC23D-G3GFP (B) with the *cis*-Golgi marker SYP31-TagRFP. Arrowheads label the ERESs. Scale bars = 20 μm.

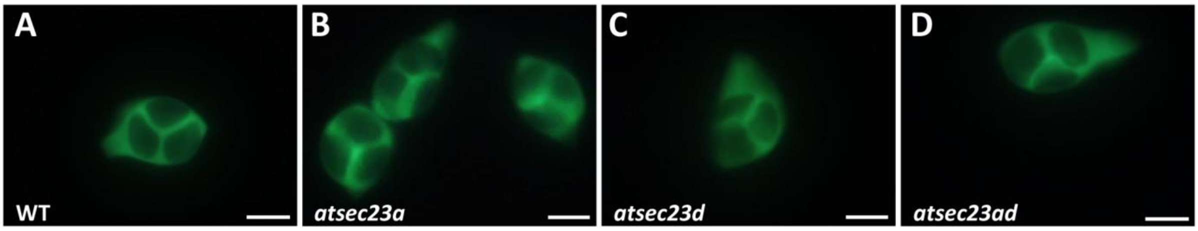

Supplementary Fig. S8. Aniline blue staining of microspores at the tetrad stage. Aniline blue staining at the tetrad stage of WT, *atsec23a*, *atsec23d*, and *atsec23ad*. Scale bars = 10 µm.

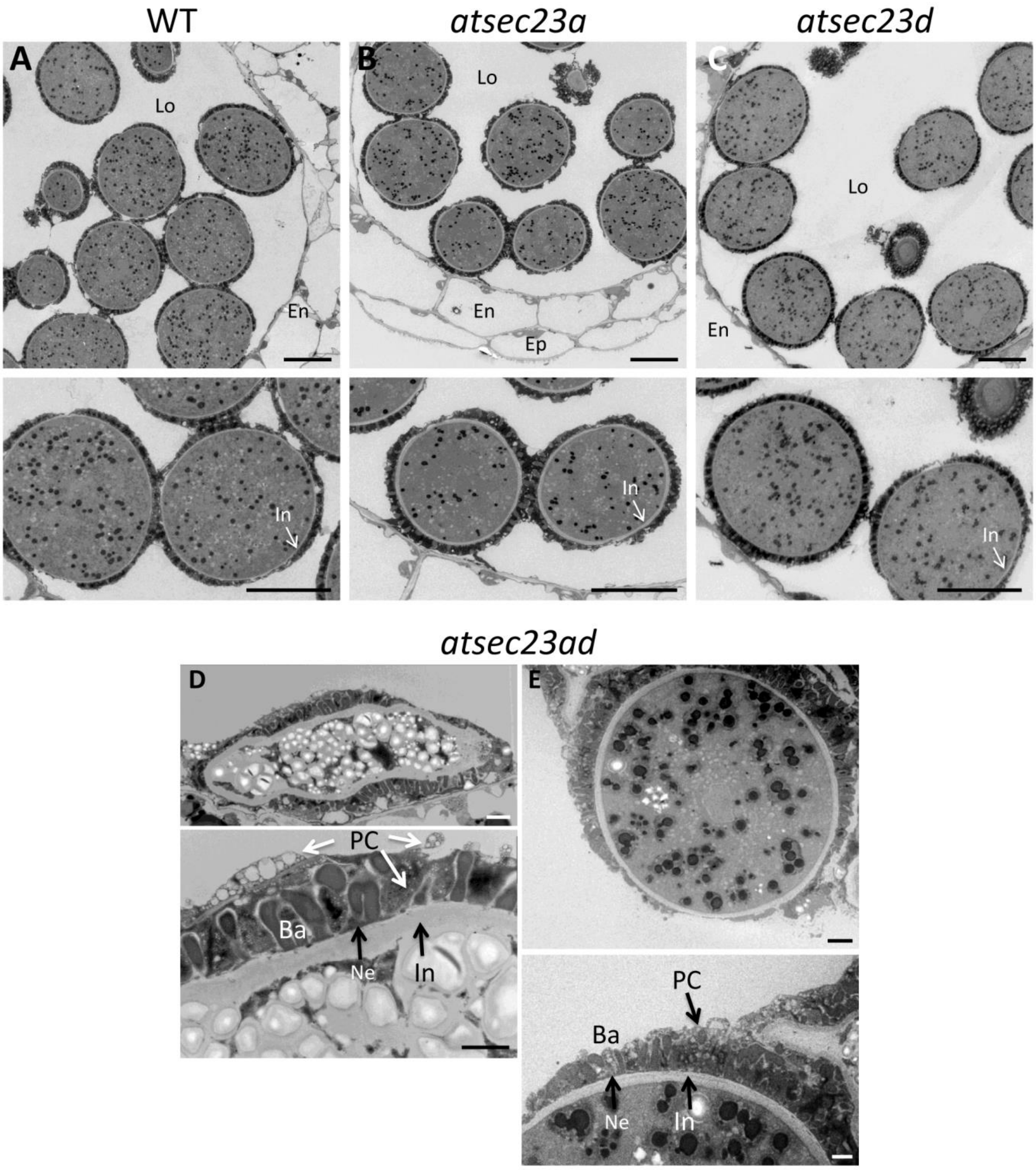

Supplementary Fig. S9. Abnormal thickening of the intine in *atsec23a* pollen grains and defective wall development in *atsec23ad* pollen grains. (A-C) SEM micrographs comparing intine of WT (A), *atsec23a* (B), and *atsec23d* (C) mature pollen grains at the tricellular stage. (D, E) SEM micrographs showing defective walls of *atsec23ad* pollen grains at the tricellular stage. Lower panels are magnifications of pollen-surface structures in the upper panels. Ba, baculum; En, endodermis Ep, epidermis; In, intine; Lo, locule; Ne, nexine; PC, pollen coat. Scale bars = 10 μm in (A-C), 2 μm in (D, E: upper panels), and 1 μm in (D, E: lower panels).

Supplementary Movie S1. Time-lapse confocal imaging of *N. benthamiana* leaf epidermal cells co-expressing AtSEC23A-G3GFP and the ERES marker AtSEC24A-TagRFP. Scale bar = 25 µm.

Supplementary Movie S2. Time-lapse confocal imaging of *N. benthamiana* leaf epidermal cells co-expressing AtSEC23D-G3GFP and the ERES marker AtSEC24A-TagRFP. Scale bar = 25 µm.
